# The RNAi Machinery in the Fungus *Fusarium fujikuroi* Is Not Very Active in Synthetic Medium and Is Related to Transposable Elements

**DOI:** 10.3390/ncrna10030031

**Published:** 2024-05-16

**Authors:** Javier Pardo-Medina, Tim A. Dahlmann, Minou Nowrousian, M. Carmen Limón, Javier Avalos

**Affiliations:** 1 Departamento de Genética, Facultad de Biología, Universidad de Sevilla, 41012 Seville, Spain; jpardo6@us.es; 2 Department of Molecular and Cellular Botany, Ruhr-University Bochum, ND 7/176 Universitätsstr. 150, 44780 Bochum, Germany; tim.dahlmann@alumni.ruhr-uni-bochum.de (T.A.D.); minou.nowrousian@rub.de (M.N.)

**Keywords:** sRNA, RNA interference, *Fusarium fujikuroi*, *Fusarium oxysporum*, high-throughput sequencing, transposable elements, Dicer gene

## Abstract

Small RNAS (sRNAs) participate in regulatory RNA interference (RNAi) mechanisms in a wide range of eukaryotic organisms, including fungi. The fungus *Fusarium fujikuroi*, a model for the study of secondary metabolism, contains a complete set of genes for RNAi pathways. We have analyzed by high-throughput sequencing the content of sRNAs in total RNA samples of *F. fujikuroi* grown in synthetic medium in the dark or after 1 h of illumination, using libraries below 150 nt, covering sRNAs and their precursors. For comparison, a parallel analysis with *Fusarium oxysporum* was carried out. The sRNA reads showed a higher proportion of 5′ uracil in the RNA samples of the expected sizes in both species, indicating the occurrence of genuine sRNAs, and putative miRNA-like sRNAs (milRNAS) were identified with prediction software. *F. fujikuroi* carries at least one transcriptionally expressed Ty1/copia-like retrotransposable element, in which sRNAs were found in both sense and antisense DNA strands, while in *F. oxysporum* skippy-like elements also show sRNA formation. The finding of sRNA in these mobile elements indicates an active sRNA-based RNAi pathway. Targeted deletion of *dcl2*, the only *F. fujikuroi* Dicer gene with significant expression under the conditions tested, did not produce appreciable phenotypic or transcriptomic alterations.

## 1. Introduction

Small non-coding RNAs (sRNAs) play very diverse regulatory roles in most biological systems, often interfering with the expression of other genes, a phenomenon known generically as RNA interference (RNAi). Among them, microRNAs (miRNAs) stand out as a large family of sRNAs with conserved regulatory functions in eukaryotes [1], usually involved in the suppression of gene expression through sequence-specific messenger RNA degradation, translational repression, or transcriptional inhibition [2]. RNA co-suppression was initially described in plants [3], but fungi have played an important role since the pioneering discovery of quelling in *Neurospora crassa* [4], and the subsequent finding of other RNAi mechanisms mediated by sRNAs in this and other fungi [5,6].

Fungal RNAi pathways show considerable plasticity and have evolved to regulate very diverse cell activities [7]. In most cases, they share the core components, which are conserved in other kingdoms. Fungal sRNAs derive from double-strand RNA precursors which are recognized and processed by a Dicer-like RNase III. These sRNA duplexes, typically 20–25 nt, are subsequently incorporated into an Argonaute (Ago) protein. Ago functions as an sRNA-guided endonuclease, recognizing target mRNAs or DNA *loci* by sequence complementarity with the incorporated sRNA, usually involved in tran-scriptional or post-transcriptional gene silencing. In some cases, an RNA-dependent RNA polymerase (RdRP) generates a double-stranded RNA (dsRNA) from a single-stranded RNA by de novo primer-independent second-strand synthesis using sRNAs as primers. The resulting RNA molecules can be complementary to target mRNAs, which act as inducers or amplifiers of the RNAi signal [2]. These core components of the pathway are present in most fungal phyla but there are relevant exceptions, such as the subphylum *Saccharomycotina* [8].

Fungal RNAi-related sRNAs can be divided into two main subclasses according to their biological role: protective sRNAs and regulatory sRNAs [6]. Those in the first group are involved in genome defense, which is probably the original evolutionary function of RNAi systems. These sRNAs include those involved in the phenomenon known as “quelling” in *N. crassa*, the first RNAi process described in fungi, which silences highly homologous repetitive elements in the genome [4,9]. Repetitive DNA sequences, whether exogenous, such as transgenes, or endogenous, such as fungal transposable elements and ribosomal genes (rDNA), are potential sources of genome instability. The post-transcriptional gene silencing of target genes is caused by the binding of double-stranded small interfering RNAs (siRNAs) to an Ago protein that forms the RNA-induced silencing complex (RISC), which in turn recognizes and degrades target mRNAs [10,11]. Other defense responses occur specifically during the sexual cycle, such as meiotic silencing by unpaired DNA (MSUD) in *N. crassa* [12] or the sex-induced silencing (SIS) in *Cryptococcus neoformans* [13]. MSUD has been also described in *Gibberella zeae* [14]. Other defense sRNAs act as antivirals that function as an innate immune system in fungi, such as those studied in *Cryphonectria parasitica* and *Fusarium graminearum* [15]. Primary sRNAs found in *Schizosaccharomyces pombe*, which govern the formation of heterochromatin in centromeric regions and appear to play an essential role in the evolution of the centromere, are also related to repetitive sequences [16].

Regulatory sRNAs, basically distributed in small interfering RNAs (siRNAs) and microRNAs (miRNAs), make up a vast network in plants and animals that plays a fundamental role in the control of gene expression [1]. Their discovery in fungi started with miRNAs and other regulatory sRNAs in *N. crassa* [17] and *Mucor circinelloides* [18]. Those of *N. crassa*, termed miRNA-like small RNAs (milRNAs), are subdivided into four classes depending on the RNAi components required for their biogenesis [6] and are derived from single-strand RNA precursors that form a typical hairpin structure. Its dsRNA region is processed to generate two complementary sRNAs; one of them is predominantly conserved, while the other is degraded. Typically, milRNAs present a strong bias for uracil bases at their 5′ termini [17]. milRNAs have been described in a wide variety of fungi, are not highly conserved, and their precursors may differ in their origin and processing [1,19]. milRNAs have not yet been identified in *M. circinelloides* and related fungi, but this species accumulates a wide variety of sRNA molecules generated by canonical and non-canonical RNAi pathways. The genesis of siRNAs is Dicer-dependent, most of them derived from exons (ex-siRNAs), while RdRP-dependent degraded RNAs (rdRNAs) are Dicer-independent and are generated by an RdRP, and correspond to the non-random degradation products of specific mRNA molecules capable of controlling mRNA levels [2,20].

Examples of sRNAs, including milRNAs and ex-siRNAs, have been found in *Fusarium* or close species. milRNAs such as those discovered in *N. crassa* have been described in *T. reesei* [21], *F. oxysporum* [22], and *F. graminerarum* [23]. In *Trichoderma atroviride*, a fungus with industrial and agronomic applications, the deletion of the different genes that encode Ago, Dicer, and RdRP produces aberrant conidia and vegetative growth [24]. Different components of the RNAi machinery are also involved in the formation of sexual spores after mating in *F. graminearum* [23], as well as in conidiation, virulence, and deoxynivalenol production [25]. In addition, a specific milRNA was found to regulate biotin synthesis [26] in this species. In *F. oxysporum,* a widely used model of phytopathogenesis, one of the Ago proteins has been described to be involved in virulence [27].

Our group focuses on the regulatory mechanisms of secondary metabolism in the gibberellin-producing fungus *Fusarium fujikuroi*, known for its complex secondary metabolism [28]. Previous data in other *Fusarium*/*Gibberella* species, and the presence in *Fusarium* genomes of genes of machinery involved in miRNAs processing, suggest the involvement of sRNAs in regulatory processes in this fungus. The aim of this work is to study the presence of sRNAs in *F. fujikuroi* by global sequencing analysis and its comparison in a parallel study with *F. oxysporum*. The results indicate the presence of sRNAs but do not support a relevant regulatory role under the culture conditions investigated, except for the presumed attenuation of the activity of some transposable elements. In further support, we deleted the only Dicer gene that showed appreciable expression under the same culture conditions, resulting in no detectable phenotypic or transcriptomic alterations.

## 2. Results

### 2.1. Small RNA Machinery in F. fujikuroi

**Figure 1:**
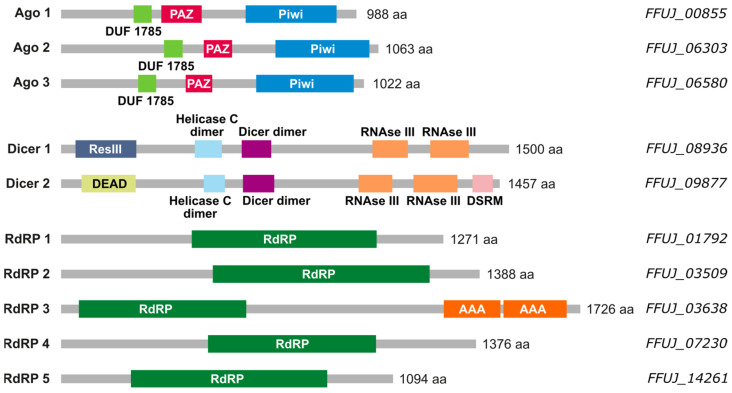
Protein domains of the potential components of the RNAi machinery in *F. fujikuroi*. The gray bars correspond to the complete sequence of the predicted protein and expected numbers of amino acids (aa) are indicated on the right. Domains represented as colored boxes are: DUF1785 (green, domain of unknown function); PAZ (red, Piwi/Argonaute/Zwille); PIWI (blue); ResIII (dark blue, type III restriction enzyme res subunits); HelC (clear blue, conserved C-terminal helicase domain); Dicer dimer (purple, Dicer dimerization domain); RNAse III (yellow, ribonuclease domain III); DEAD (pale green, domain of the DEAD-like helicase superfamily); DSRM (pink, double-stranded RNA binding motif); RdRP (green, RNA-dependent RNA polymerase); AAA (orange, AAA domain, from ATPase associated with various cellular activities).

As a first step to study the existence of miRNAs in *F. fujikuroi*, the occurrence of genes for the expected enzymatic machinery in its genome was analyzed. The orthologous genes of the corresponding proteins described in *N. crassa* were used as reference. The genes *qde-2* [4,9], a member of the Argonaute family, *dcl-1* and *dcl-2*, members of the RNAse III family, and *qde-1*, an RdRP, were used as input to perform BLAST searches for orthologs in the *F. fujikuroi* genome. As a result, ten genes were found: three genes belonging to the Argonaute family, *FFUJ_00855*, *FFUJ_06303*, and *FFUJ_06580*, denominated *ago1*, *ago2*, and *ago3*; two RNAse III genes, *FFUJ_08936* and *FFUJ_09877*, named *dcl1* and *dcl2*; and five genes corresponding to RdRPs, *FFUJ_01792*, *FFUJ_03509*, *FFUJ_03638*, *FFUJ_07230*, and *FFUJ_14261*, in turn *rdr1–5*. Subsequently, their sequences were analyzed using the PFAM protein domain database [29] and the results are depicted in Figure 1.

The three Ago proteins of *F. fujikuroi* have all the known domains of the Argonaute family (DUF1785, PAZ, and Piwi). The two Dicer proteins also have typical features of the RNAse III family. The five putative RdRPs have their characteristic domain; it is worth noting that *FFUJ_03638* also presents ATPase domains. The presence of complete machinery led us to perform a global analysis of the presence of small RNAs in *F. fujikuroi*. To verify its prevalence in *Fusarium*, taking advantage of the previous availability of total RNA samples, the analysis was extended to *F. oxysporum*.

### 2.2. Sequencing and Characterization of Small RNAs in F. fujikuroi and F. oxysporum

Total RNA samples of wild-type strains of *F. fujikuroi* and *F. oxysporum* from a previous transcriptomic analysis [30] were used to search for sRNA. Mycelia grown for 3 days in darkness were illuminated for 1 h or kept in darkness. Therefore, the RNA extraction method and quality parameters were as previously described. Illumination for 1 h was chosen because it was the maximum response time to the upregulation of photoinduced gene mRNAs previously investigated in both fungi [31,32]. RNAs from two samples of each condition with a size below 150 nt, covering sRNAs and their precursors, were used to construct the sRNA libraries.

To find all miRNA-like RNAs, total reads of samples of each species from both culture conditions were merged into a single file. The sRNAs were mapped to the genome of the respective species so that reads that occurred several times within the dataset were collapsed to a single entry and their read count was added to the fasta header, decreasing file size without losing information. The alignment results of the collapsed reads are shown in Supplementary Table S1.

As an additional benefit of using collapsed reads, the overestimation of over-represented reads, such as those originating from tRNAs and rRNAs, is decreased (Supplementary Table S2). However, while redundant reads showed only a small proportion in the merged reads of *F. fujikuroi* (8.5%), they were predominant in *F. oxysporum* (82.5%), indicating that an over-represented amount of rRNAs and tRNAs was incorporated during the preparation of the *F. oxysporum* library. This was checked by mapping the *F. fujikuroi* and *F. oxysporum* reads to the tRNA and rRNA sequences, which allowed the discarding of the tRNA- and rRNA-derived repetitive sequences (details in Section 4.3).

**Figure 2:**
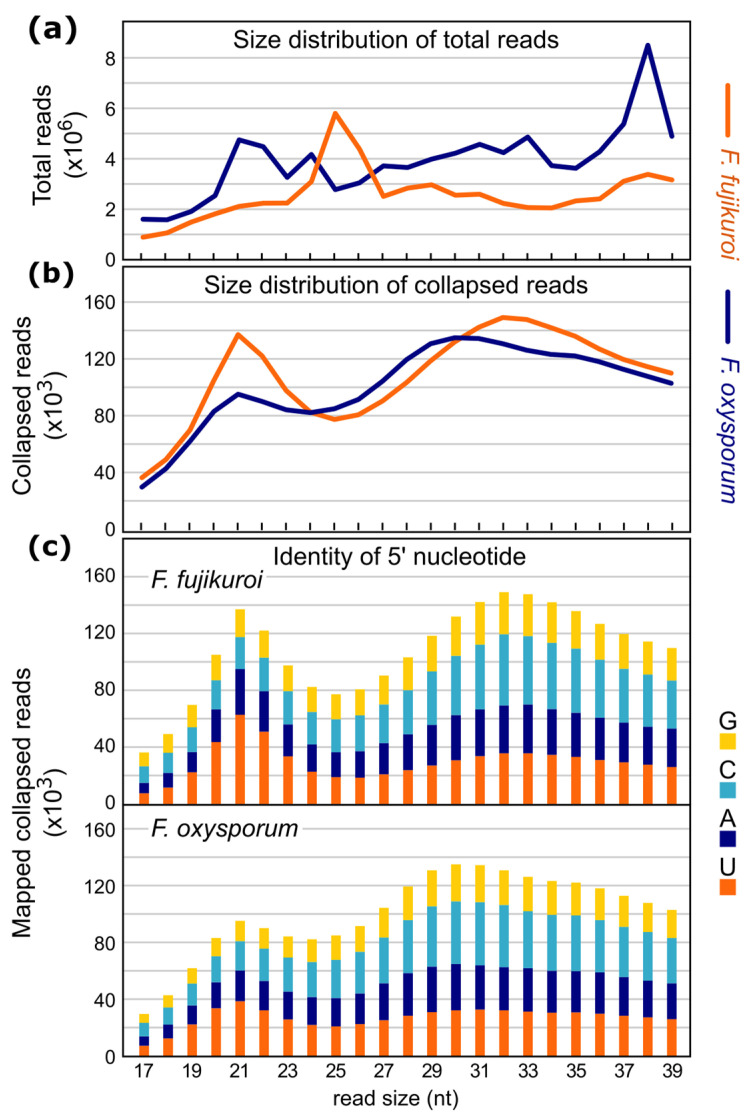
Characterization of mapped sRNAs. (**a**) Size distribution of all mapped reads within the merged datasets of *F. fujikuroi* and *F. oxysporum*. (**b**) Size distribution of all collapsed reads within the merged datasets of both fungi. A peak at 19–22 nt was observed in *F. fujikuroi*, while a second peak was present between 28 and 35 nt in both fungi. The peak at 19–22 nt is typical for Dicer-processed small RNAs. (**c**) Size distribution and 5′ nucleotide preference of sRNAs in *F. fujikuroi* and *F. oxysporum*. sRNAs with a size of 19 to 23 nt predominantly started with a 5′ uracil (up to 45.8% in *F. fujikuroi* and 40.5% in *F. oxysporum*), while sRNAs of other sizes showed balanced proportions for each of the four nucleotides. The peak between 19 and 23 nt was higher in *F. fujikuroi* than in *F. oxysporum*.

Dicer-like RNases III process many fungal sRNAs, such as siRNAs and the majority of milRNAs. It was shown that sRNAs processed by Dicer-like endonucleases show a strong preference for a size of 19 to 22 nt and a 5′ uracil [17,33,34]. Thus, size distribution and 5′ nucleotide preference analyses of mapped total and collapsed sRNA reads were performed (Figure 2a,b). To elucidate the existence of ‘true’ sRNAs, the proportion of the four nucleotides at the first position was analyzed for the collapsed reads (Figure 2c). The results showed an increased proportion of 5′ uracil in the expected sizes RNA samples for both species.

### 2.3. Origin of sRNAs in F. fujikuroi and F. oxysporum

The increased amount of sRNAs starting with uracil is consistent with the presence of small interfering RNAs (siRNAs). Functional RNAi machinery was described in *F. graminearum* [35]; therefore, it may also be present in *F. fujikuroi* and *F. oxysporum*. If sRNA originates from double-stranded RNAs, which are produced by RNA-dependent RNA polymerases as a counter-reaction to retrotransposons or to other endogenous transcripts, we should be able to identify transcribed *loci* that produce sRNAs from both DNA strands.

**Figure 3:**
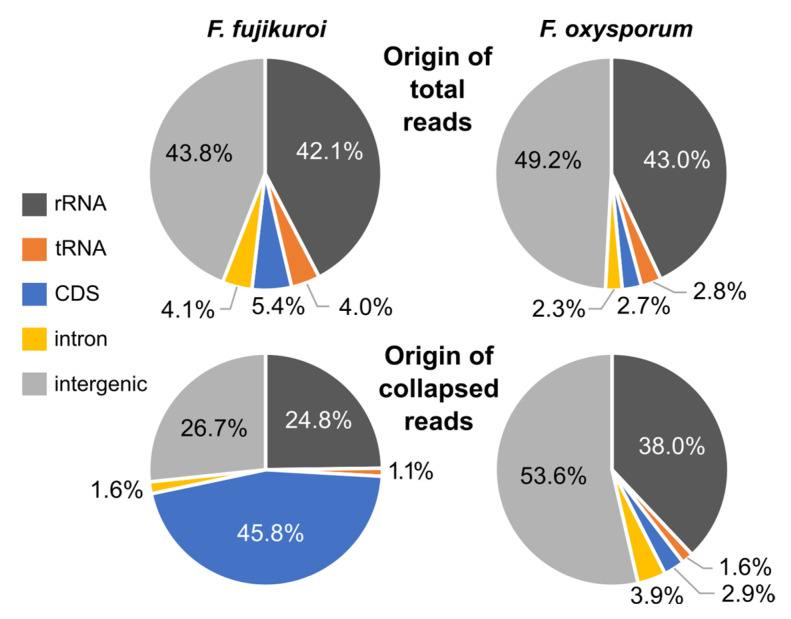
Origin of total and collapsed reads (x ≥ 10) in *F. fujikuroi* and *F. oxysporum*. CDS: coding sequence.

The amounts of sRNAs originated either from different parts of a gene or from multiple copy genes (rRNA and tRNA) were very similar in *F. fujikuroi* and *F. oxysporum* (Figure 3). Only the numbers of collapsed reads (x ≥ 10) mapping on the sense and antisense strands at coding sequences (CDS) (marked by red in Supplementary Table S3) varied strongly in the datasets. Interestingly, in *F. fujikuroi* the number of collapsed reads (x ≥ 10) mapping to CDS *loci* was significantly higher compared to those in *F. oxysporum*. This agrees with the findings of the length and nucleotide distribution of the collapsed reads (Figure 2). It must be noted that the high number of collapsed reads mapping to CDS *loci* in both fungi could also be due to nonspecific mRNA-degradation.

### 2.4. sRNAs Related to Transposable Elements

Transposable elements are often posttranscriptionally silenced. BLAST analyses of sequences from the transposable elements skippy, Ty1/copia, and impala from *F. oxysporum*, HobS from *F. fujikuroi*, and MAGGY from *Magnaporthe grisea* were used to identify siRNAs that could be generated in response to these mobile genetic elements. The corresponding annotated proteins and genomic sequences were used for blastp and tblastx analyses, and the results are displayed in Table 1.

**Figure 4:**
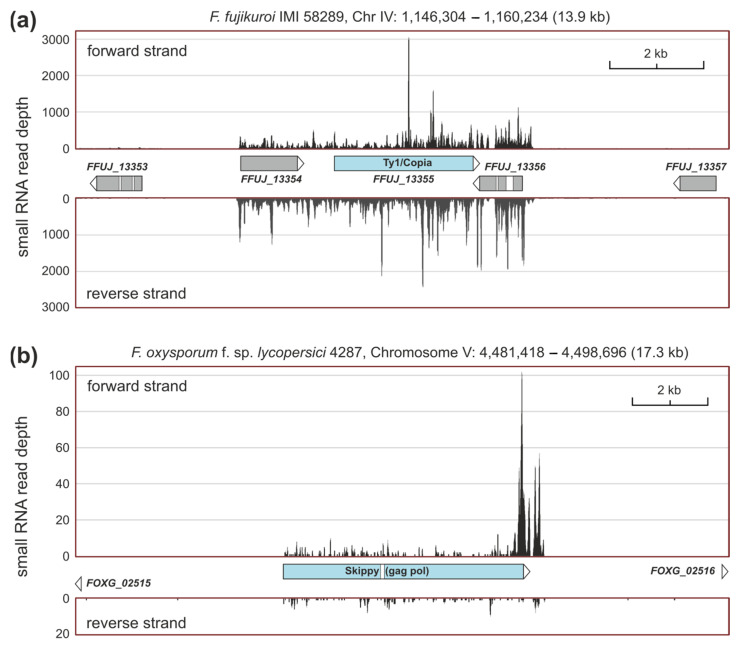
Accumulation of sRNAs in retrotransposons. (**a**) In the copia in *F. fujikuroi.* (**b**) In the skippy retrotransposon in *F. oxysporum*. Samtools representation of small RNA mapping results under dark and light. Read counts of forward and reverse strands are shown. Differences in read counts for *F. fujikuroi* and *F. oxysporum* can be explained by weaker expression and/or lower total read counts in *F. oxysporum*.

Both *Fusarium* species contain transposable elements in their genomes, but this does not mean that they are active. *F. fujikuroi* carries at least one transcriptionally expressed copy of a Ty1/copia-like element, which is surrounded by two genes that encode uncharacterized proteins. In all three *loci*, most especially in the retrotransposon sequence, sRNAs were present in their sense and antisense DNA strands, a clear indication of an active siRNA-based RNAi pathway (Figure 4a). In *F. oxysporum*, only skippy-like elements seemed to be slightly expressed in the growth conditions studied and could be silenced by RNAi. A nonannotated example is shown in Figure 4b.

### 2.5. Differentially Expressed sRNA-Producing Loci under Dark and Light

To find sense and antisense sRNAs that can be differentially synthesized during growth in darkness and after illumination, each sample was aligned separately with the reference genome. The read counts for each dataset were calculated with the R script summarizeOverlaps, which is part of the Bioconductor GenomicAlignments package. After normalization, the features that showed the differential formation of small RNAs were calculated with DESeq2 (adjusted *p*-value < 0.1). The sense strand was used as a control for the impact of altered conditions on gene expression.

**Figure 5:**
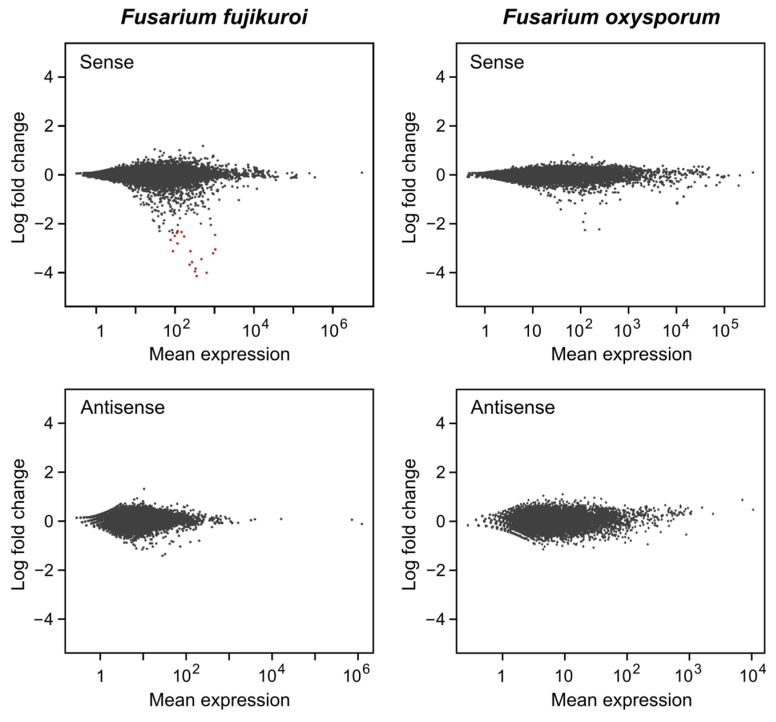
Log-fold change and sRNA mean expression of sRNA-producing protein-encoding genes of *F. fujikuroi* and *F. oxysporum.* sRNAs generated in the sense and antisense orientation to the corresponding genomic features are indicated. Genes that show differential formation of sRNAs in dark vs. light are highlighted by red dots.

In the comparison of dark vs. light samples in *F. fujikuroi*, 18 genes were found to differentially form sRNAs under both conditions (Supplementary Table S4). Because of the absence of antisense sRNAs, these strand-specific sRNAs are very likely derived from the unspecific degradation of single-stranded mRNA. In fact, these genes were among the most light-induced in the original mRNA data [30]. Using the same analysis of sRNAs that originate from the antisense strand, either by transcription of the antisense loci or by formation of double-stranded RNAs by RdRPs, no sRNA-producing loci were found showing the differential expression of sRNA under the tested conditions. Plots of the log-fold change (FC) of the *F. fujikuroi* genes and their sRNA average expression are shown in Figure 5.

The same calculation was also performed for *F. oxysporum*. No differentially expressed sRNA-producing loci were found in the sense or antisense strand that matched the criteria of log2 ± 1 and the adjusted *p* value < 0.1. Plots of the log2 fold change in genes from *F. oxysporum* and their sRNA mean expression are shown in Figure 5.

### 2.6. Prediction of milRNAs by miRDeep2

As a more stringent method for the detection of genuine milRNAS, we applied the miRDeep2 algorithm. As a result, very few de novo predicted putative miRNA-like RNAs were detected, most of them with a low miRDeep2 score (<5) or without showing the characteristic precursor formation, known from fungal milRNAs. In comparison with previous studies in fungi, only a few predicted milRNAs seemed “true” products of microRNA-like Dicer-dependent processing. Putative milRNAs from *F. fujikuroi* are described in Supplementary Table S5 and Table 2.

The locations of the putative milRNA sequences usually coincide with the ORFs or promoter regions of annotated genes (Supplementary Figure S1a), which could be regulatory targets. Most of these genes have low expression levels (Supplementary Figure S1b), making it unlikely, in the case of the same transcriptional orientation, that they are mRNA degradation products. Moreover, some of them (milRNAs #1, 5, 6, 9, 10, and 11, Table 2) are transcribed antisense to the associated gene, suggesting that the corresponding milRNAs could bind to the corresponding mRNAs and interfere with their function. A striking case is that of potential milRNA #8, particularly abundant and located in a subtelomeric region at more than 3 kb from the 3′ end of the closest gene. The sequence of the 21 bases of this putative milRNA contains 3.5 copies of the UUAGGG telomeric DNA repeat, suggesting a regulatory role in telomere elongation in this fungus. Notably, such repeats are absent in the rest of the precursor RNA sequence, strongly pointing to a telomeric-related function for this milRNA.

To identify possible regulatory targets for the potential milRNAs described in Table 2, excluding the putative telomere-related #8 milRNA, their sequences were subjected to BLAST analysis against the genome of *Fusarium fujikuroi* IMI58289. Except the original locus, no targets corresponding to the complete sequence of any of the 10 milRNAs were found, but in some cases coincidences were found that exceeded what could be explained by chance. Attention was paid to those with an *e*-value lower than 1, which are described in Table 3. They include sense and antisense targets. One of them (#8) is mostly coincident with an internal ribosomal RNA sequence, suggesting a possible role in its regulation by pairing. Another (#7) exhibits considerable antisense orientation identity with the genes for three of the putative transposable elements described in Table 1, which could be inactivated by the binding of this milRNA to mRNA encoded by these genes.

### 2.7. Generation and Phenotypic Characterization of Δdcl2 Mutants

**Figure 6:**
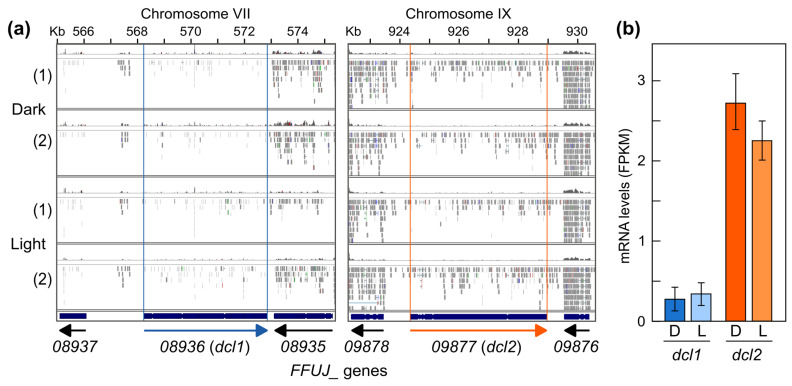
Expression of the *dcl1* and *dcl2* genes in wild-type *F. fujikuroi* IMI58289 grown in DG minimal medium in the dark or after 1 h of illumination according to available RNA-seq data [30]. (1) and (2): biological replicates. (**a**) Transcript readings in the genomic region of genes *dcl1* (left panel) or *dcl2* (right panel) represented on the same scale using the IGV software (Integrative Genomics Viewer, version 2.16). (**b**) Transcript levels (FPKM) of the *dcl1* and *dcl2* genes derived from these data.

The RNA-seq data available from the total RNA samples used for the sRNA analysis allowed us to verify the transcription levels of the two Dicer genes identified in the *F. fujikuroi* genome. The results showed that, under the experimental conditions used, the expression level was low in the case of *dcl2* and barely detectable in the case of *dcl1* (Figure 6). Therefore, to gain more information about the role of milRNAs in *F. fujikuroi* under these conditions, we investigated the effect of deleting the *dcl2* gene. For this purpose, the *dcl2* ORF was replaced by a hygromycin resistance cassette. As a result, two Δ*dcl2* mutants, named SG293 and SG294, were obtained (the procedure and molecular characterization are described in Section 4.4). On the contrary, attempts to delete *dcl1* were unsuccessful, suggesting a lack of accessibility of the recombination machinery to this 5 kb DNA region.

The phenotype of SG293 and SG294 mutants was compared with that of the wild type in different culture media, liquid or solid, with different sources and amounts of nitrogen and at different pHs. Incubations were carried out at 22 °C or 30 °C in darkness or under illumination. No differences between the wild type and the two Δ*dcl2* mutants were observed, including growth rate, mycelial development, morphology, or pigmentation, under any of the conditions tested. Their conidiation capacities were also evaluated, but no significant differences were found between the strains. Representative examples of the aspects of the colonies under different culture conditions and conidiation data are shown in Supplementary Figure S2.

### 2.8. RNA-Seq Analysis of a Δdcl2 Mutant

Additionally, the effect of the deletion of *dcl2* in the transcriptome was investigated by RNA-seq analysis of SG294 under the culture conditions used for the analysis of sRNAs. The readings obtained and their basic characteristics are described in Supplementary Table S6. The representation of the expression data corrected for each gene and expressed as RPM (reads per million mapped reads) in a bean plot graph (Supplementary Figure S3a,c) showed a high parallelism between all the samples. The data store similarity tree (Supplementary Figure S3b) showed some experimental dispersion only in the case of Replicate 2.

The DESeq2 tool [36], implemented in SeqMonk, which requires raw counts for quantification, was used to compare between conditions. Scatter plot representations of the wild-type and Δ*dcl2* transcriptomes showed few discrepancies (Supplementary Figure S3d). This includes the genes in which the milRNA sequences are located (Table 2, Supplementary Figure S4), as well as the possible gene targets described in Table 3 (Supplementary Figure S5a), for which there were no appreciable differences between the wild type and the Δ*dcl2* mutant. The selection of differentially expressed genes based on criteria combining a log2 fold change of 1 and a *p*-value of 0.05 revealed that only four genes were considered deregulated among the two strains: *FFUJ_09878*, *FFUJ_09877*, *FFUJ_09875*, and *FFUJ_14259*. The gene *FFUJ_09877* corresponds to the *dcl2* gene, while *FFUJ_09878* and FFUJ_09875 are adjacent to the deletion, so their overexpression in the mutant can be explained by a side effect of the HygR cassette in the *dcl2* locus. *FFUJ_14259* encodes a putative GTP cyclohydrolase I and was the only gene overexpressed in the mutant and not close to the deletion locus (log2 FC = 3.7). When an alternative algorithm like EdgeR was used, another gene fell into the activated category, *FFUJ_14373* (log2 FC = 5.6), which encodes an uncharacterized protein.

The lack of *dcl1* expression under the culture conditions investigated strongly suggests that the identified sRNAs mainly derive from Dcl2 activity. Considering the role that sRNAs can play in silencing transposable elements, as deduced from the accumulation of sRNAs in some of them or from their presence among possible milRNA targets, the expression of annotated genes from putative transposons (Table 1) was checked in the RNA-seq data of the wild-type strain and the Δ*dcl2* mutant. Most of them showed very low levels of transcripts, and these levels were not significantly altered by *dcl2* deletion (Supplementary Figure S5b). The lack of effect of *dcl2* deletion suggests that transposon-associated sRNAs exert their function posttranscriptionally on mRNAs produced by these transposons, as appears to be the case at least for some Ty1/copia elements, possibly affecting their availability for translation.

## 3. Discussion

We have used sRNA profiling as a starting point to understand the relevance of RNAi in *F. fujikuroi*. Previous evidence already indicated RNAi processes in *F. graminearum* [35], with regulatory roles in sexual and asexual reproduction, secondary metabolite production, and virulence [25,37], and in *F. oxysporum* [22], with increasing evidence of their involvement in virulence [27,38,39]. The genome of *F. fujikuroi* contains a complete set of genes for the RNAi pathway, including those for two Dicer proteins, like those commonly found in ascomycetes, five RdRPs, as described in *F. graminearum* [25], and three Ago proteins, one more than in this species. Here, we provide clues about the functioning of an RNAi pathway in *F. fujikuroi*. In massive sRNA sequence data, the preference for a 5-prime uracil found for sRNAs within the size range of 20 to 22 nt in *F. fujikuroi* is a strong indication for Dicer-processed sRNAs. This conclusion was supported by the enrichment of these sRNAs in *F. oxysporum*. The sRNAs originated in both species from diverse genomic *loci*, with a remarkable proportion of rRNAs. This is consistent with previous results in *F. oxysporum*, suggesting that qiRNAs, a class of DNA damage-induced sRNAs derived from rDNA *loci*, may also be present in both fungi [22,40].

Indications of Dicer-dependent siRNAs are the large number of antisense reads to transposable elements and the identification, using the demanding miRDeep2 algorithm, of a putative milRNA capable of binding to three transposon sequences. Quelling-like RNAi pathways and their role in limiting transposon and retrotransposon activity, maintaining genome integrity during vegetative growth, have been confirmed in fungal models, such as *A. nidulans* [41], *T. atroviride* [24], or *F. graminearum* [35]. To investigate whether sRNAs are produced in response to selfish genetic elements, such as transposons, BLAST analysis of *Fusarium* transposable elements was performed to identify those *loci*. Interestingly, Ty1/copia elements showed a strong accumulation of sRNAs in their sequences in the antisense orientation in *F. fujikuroi*. In *F. oxysporum,* this might also be the case for skippy-like elements. Furthermore, a possible antisense milRNA was identified in *F. fujikuroi* with sequence identity to three putative Ty1/copia transposon genes. Although evidence for a functional RNAi mechanism in *F. oxysporum* has been provided before [42], it might be interesting to further analyze whether there is a downregulation of transcriptionally active transposable elements in *F. fujikuroi* through mRNA-control mechanisms.

Our study also addressed the possible effect of light on sRNA production in the investigated fungi. The direct involvement of sRNAs in light regulation has not been solidly established in fungi. In *T. atroviride*, light-dependent asexual reproduction was affected in RNAi pathway mutants. Apparently, this was not due to regulatory effects on light perception genes, but to those directly involved in the morphogenetic changes required to produce conidia, which were a direct target of Dicer2-dependent sRNAs produced under illumination [24]. Similarly, *dicer* and *ago* mutants of *Metarhizium robertsii* showed reduced abilities to produce conidia in light [43]. In the *Fusarium* genus, there was evidence of RNAi involvement in conidiation in *F. graminearum*, but it was only detected with low light intensity [25]. Thus, some sRNAs could be involved in the photoregulation of conidiation in this species. The study of the differential formation of sRNAs in dark versus light samples that we have carried out in *F. fujikuroi* and *F. oxysporum* was an attempt in this direction. In *F. fujikuroi*, only 18 genes showed the formation of sRNAs in the sense orientation, but not in the antisense orientation, while in *F. oxysporum* no regions matching these criteria were found in either the sense or the antisense DNA strand. Most of the 18 genes are known to be upregulated by light [30], and their sRNA profiles did not target specific sequences but random locations along highly expressed genes. Thus, it is very likely that these sRNAs originated from mRNA processing and/or fragmentation, suggesting the lack of involvement of sRNA in light regulation.

The prediction of milRNAs in both fungi using the miRDeep2 tool revealed several putative candidates. Their prediction scores were low, and both the read counts and the precursor structures suggested that in some cases they might be false positives. However, the characteristics of at least several of them suggest that they are genuine milRNAs with potential regulatory functions, especially those that correspond to antisense sequences with respect to putative target genes. Two of them deserve special mention for their possible implications in telomeric and rRNA regulation. In addition, BLAST searches against the *Oryza sativa* genome*,* of which *F. fujikuroi* is a pathogen, revealed instances of striking sequence matches with some plant genes. The best match was that of milRNA #11 with an internal 18 bp sequence of a chalcone synthase-like gene (XM_015774125), with an e-value of 0,18. Other matches were shorter (16 bp coincidence), but could be biologically relevant, as found for milRNA #3 with two rice genes, one encoding an Ethylene Insensitive 3-like 1 protein (XM_015791088), a transcription factor linked to the ethylene stimulus wound signaling response, and another encoding a cyclin-C1-1-like protein (XM_015756276). Certain fungi, such as *Botrytis cinerea*, can transfer siRNAs during infection to the plant host and sequester components of the plant RNAi pathway to suppress the expression of host immunity genes [44]. These sRNAs have also been found in extracellular vesicles that function as vehicles for sRNA exchange between species [45]. In *F. graminearum*, Fg-sRNA1 can suppress the wheat defense response by targeting and silencing a resistance-related gene (TaCEBiP) [46]. In addition, different miRNA target genes of *F. graminearum* have been identified in *Hordeum vulgare* and *Brachypodium distachyon*, whose expression is interfered after fungal infection, weakening the defense response of plants [37], and an miRNA interplay has been recently found to play key roles in the pathogenesis of maize by *Fusarium verticillioides* [47]. It is a tempting hypothesis that a major function of the sRNA-forming machinery of *F. fujikuroi* is to interfere with the expression of some critical rice genes during the infection process.

To verify the relevance of sRNAs in *F. fujikuroi*, we focused our attention on the two dicer genes (*dcl*) identified in its genome. Interestingly, only one of them, *dcl2*, was appreciably expressed under the culture conditions investigated, and deletion mutants could only be obtained for this gene, a fact possibly related to the extremely low expression of *dcl1*. However, no phenotypic alterations could be observed in two independent Δ*dcl2* mutants. In contrast, Δ*dcl1* and Δ*dcl2* mutants have been described *in F. graminearum*, and both were affected in conidiation (in liquid medium and under special light conditions), ascospore discharge, and aurofusarin and deoxynivalenol synthesis, and in the case of the *Fgdcl1* mutant, also in wheat spikes infection [25]. However, no effect was observed for conidiation in the Δ*dcl2* mutant of *F. graminearum* on solid medium under constant illumination. In our case, *F. fujikuroi* IMI52829 does not produce conidia in submerged cultures, preventing comparison with *F. graminearum*. In the case of secondary metabolites, *F. fujikuroi* lacks gene clusters for the synthesis of tricothecene or aurofusarin, but no changes in the production of other colored metabolites, such as bikaverin, were appreciated. Therefore, at least in terms of conidia production and visually detected secondary metabolites, the results indicate differences in Dcl2 functions between *F. fujikuroi* and *F. graminearum*, reflecting varying regulatory demands in the two species.

## 4. Materials and Methods

### 4.1. Strains and Culture Conditions

The wild-type strain of *Fusarium fujikuroi* IMI58289 was obtained from the Imperial Mycological Institute (Kew, Surrey, UK). The strains were cultured in DG medium, whose composition per liter is 30 g glucose, 3 g NaNO_3_, 1 g KH_2_PO_4_, 0.5 g KCl, 0.5 g MgSO_4_·7H_2_O, and microelements [48], solidified when required with 16 g agar. This medium was used for most purposes under the indicated conditions. For conidial production, the strains were cultured for 7 days at 30 °C under illumination on EG agar medium, whose composition per liter is 1 g glucose, 1 g yeast extract, 1 g NH_4_NO_3_, 1 g KH_2_PO_4_, 0.5 g MgSO_4_·7H_2_O, and 16 g agar. Media used for protoplast formation and transformation have been described [48].

For total RNA isolation for small RNAs sequencing and RNA-seq, 10^6^ conidia of *F. fujikuroi* were inoculated into 100 mL of DG medium in 500 mL Erlenmeyer flasks and incubated in the dark for 3 days in an orbital shaker at 30 °C and 200 r.p.m.. After this time, 25 mL samples were taken from the cultures under red safelight and transferred to Petri dishes, where they were incubated at the same temperature for 1 h in the dark or under illumination. Cultures of *F. oxysporum* were incubated for 3 days in 145 mm Petri dishes containing 80 mL of liquid DGasn medium (DG with 3 g L^−1^ asparagine instead of NaNO_3_). Mycelial samples were obtained by filtration, frozen immediately in liquid nitrogen, and stored at −80 °C.

For the phenotypic characterization of Δ*dcl2* mutants, the wild-type strain and transformants *dcl2*-3 and *dcl2*-4 were cultured on DG, DGasn, DG low nitrogen (DG with 0.3 g L^−1^ NaNO_3_), DG with neutral pH (K_2_HPO_4_ was used instead of KH_2_PO_4_), DGpep (DG supplemented with 2 g L^−1^ peptone), or PDA (potato dextrose agar). All strains were cultured in triplicate in each medium for one week at 22 °C or 30 °C, in the dark or under continuous illumination.

In all cases, illumination conditions consisted of exposure to 7 W m^−2^ white light, provided by a set of four fluorescent tubes (Philips TL-D 18 W/840) at a distance of 60 cm.

### 4.2. sRNA Sequencing and Analysis

The sRNA analysis was carried by selecting transcripts with a size <150 nt in total RNA samples used in a previously published experiment [30], in which the influence of light and CarS protein in the transcriptomes of *F. fujikuroi* and *F. oxysporum* was analyzed. They were sequenced on Illumina’s Hiseq platform in the mode of 50 bp single read by LifeSequencing S. L. (Valencia, Spain). Raw reads for all samples were trimmed, filtered, and quality controlled with AfterQC. The readings obtained and their basic characteristics are described in Supplementary Table S7. Data from both fungi were processed in a uniform manner.

The mapping of sRNA reads was performed using Bowtie v1.1.1, designed to map short Illumina reads of ~35 nt, or Bowtie2 v2.2.9 for longer reads.

The gff annotation files from both fungi were used to extract fasta files containing coding, intronic, and intergenic sequences. The extraction was performed after creating the corresponding features with the Artemis 16.0.0 genome browser.

To perform the miRDeep2 analysis, white spaces from the genome fasta files and collapsed reads shorter than 17 nt were eliminated. Analysis was carried out without any additional information on *Fusarium* milRNAs, other fungal milRNAs, or known *Fusarium* precursors. No score cutoff was used, and all precursors were analyzed.

### 4.3. Identification and Discarding of Redundant Sequences

To characterize the redundant reads of *F. fujikuroi* and *F. oxysporum,* they were mapped to tRNA and rRNA sequences. The corresponding tRNA sequences were extracted from the annotation files and the putative rRNA-producing *loci* (rDNAs) were predicted with RNAmmer v1.2 for both fungi. The number of reads discarded by Bowtie due to multiple alignments (-m 5) was higher in *F. oxysporum*, which was in agreement with a higher number of rDNAs. Rather than the higher number of reads discarded that have biological causes, the reason may be that rDNAs were not considered during assembly of the *F. fujikuroi* genome. To test this hypothesis, the collapsed reads were mapped to the predicted rDNAs of *F. oxysporum*, and the total read count was calculated. As hypothesized, most of the total reads within the *F. oxysporum* dataset could be mapped to rDNAs (73.6%). Although the dataset appeared rather diverse, the proportion of collapsed mapped reads (Supplementary Table S1) was only slightly different between *F. fujikuroi* (67.3%) and *F. oxysporum* (55.7%). Therefore, *F. oxysporum rDNA* was used for a second analysis with total reads from *F. fujikuroi* and 70.3% of the reads mapped to *Fusarium* rDNA.

### 4.4. Generation of ∆dcl2 Transformants

To replace the *dcl2* gene with a selection marker, a plasmid was constructed by homologous recombination in *Saccharomyces cerevisiae* [49] containing an hygromycin antibiotic resistance cassette surrounded by 5′ and 3′ noncoding *dcl2* sequences. Both fragments, consisting of approximately 1.5 kb of the 5′ and 3′ UTR *dcl2* ORF flanking regions, were amplified by PCR with PS1 and PS2 primer sets (these and the other primer sets mentioned below are described in Supplementary Table S8). These fragments included specific tails with homology to sequences from the HygR cassette and the plasmid pRS426 (vector database: https://www.addgene.org/vector-database/3989/, accessed on 13 May 2024). The HygR cassette was amplified by PCR with the PS3 primer set from the plasmid pCSN44 (GenBank accession LT726870). All fragments along with the linear plasmid pRS426 were incubated with competent cells of *S. cerevisiae* to obtain the desired construction, which was used to replace the *dcl2* sequence by the transformation of protoplasts from wild-type *F. fujikuroi*. The putative transformants were then selected by growth on a medium containing 100 mg L^−1^ hygromycin (Roche, Mannheim, Germany). As a result, four hygromycin-resistant transformants were obtained and purified by the selection of uninucleate spores in three successive steps.

The four transformants were subjected to a molecular analysis to determine whether *dcl2* had been replaced by the HygR cassette by homologous recombination (Supplementary Figure S6a). Several combinations of primers were used to check the absence of the *dcl2* coding sequence and the presence of the HygR marker (*hph* gene). The genomic DNA of the transformants #3 and #4 did not contain the native *dcl2* sequence and gave by PCR the expected band using primers specific for the 5′ *dcl2* sequence and the *hph* gene (Supplementary Figure S6b). As an additional verification step, Southern blot hybridization with a radioactively labeled probe was performed. Southern blot was achieved essentially as described [50]. Samples of at least 10 μg of genomic DNA were digested with the *Hind*III restriction enzyme and the resulting products were electrophoresed on a 0.7% agarose gel and subjected to the Southern blot protocol using a positively charged nylon membrane (Hybond-N from Amersham). The probe used, which corresponds to a region downstream of *dcl2* (Supplementary Figure S1), was obtained from wild-type genomic DNA by PCR with the P1.9 primer set. The probes were labeled with ^32^P dCTP and the membranes were exposed for radioactivity detection in a radioisotope imaging system (FujiFilm FLA 5100, Life Science, Cambridge, MA, USA). The results confirmed that the HygR cassette had effectively replaced the *dcl2* gene in the transformants #3 and #4 (Supplementary Figure S6c), named SG293 and SG294, respectively.

### 4.5. RNA-Seq Analyses of ∆dcl2 Mutant

Cultures of the wild-type strain and ∆*dcl2* mutant SG294 were grown in parallel, with three independent biological replicates, resulting in 6 samples being analyzed. Incubations were carried out for three days in liquid DG medium in the dark, followed by 4 h of adaptation to static culture in Petri dishes. Total RNA was extracted with Trizol (Invitrogen, Paisley, UK) from 150 to 200 mg of ground mycelia samples, using the protocol described by the manufacturer. RNA concentrations were quantified with a Nanodrop ND-1000 spectrophotometer (Nanodrop Technologies, Wilmington, DE, USA). Twenty μg of each RNA sample was treated with DNAse I in columns of the NucleoSpin RNA kit (Macherey-Nagel, Düren, Germany) following the manufacturer’s instructions. The quality and integrity of the RNA samples were evaluated by spectrophotometry (A260/A280 > 1.8 and A260/A230 > 1.5) and visualization in an agarose gel. The samples were sent for mass sequencing to LifeSequencing S. L. (Valencia, Spain). The RIN values were above 8.5 in all samples. Sequencing was carried out using the Illumina platform [51]. The samples were sequenced on Illumina’s NextSeq platform in 75 bp single-read mode. Sequences were mapped with STAR [52]. Quantitation was performed by merging transcripts and counting reads over exons and percentile normalized.

## 5. Conclusions

This work constitutes the first approach to the study of sRNA production in *F. fujikuroi* and its possible functions. Genomic and sRNA data strongly support the existence of an operative RNAi mechanism in this fungus, although in the culture conditions investigated it seems to play a secondary role, associated with keeping inactivated some possibly active transposable elements in its genome. Mutants of the only Dicer gene expressed under such conditions, *dcl2*, did not show phenotypic alterations. The possible role that RNAi mechanisms may play in the pathogenesis process or in the predictably adverse conditions that the fungus may face in nature remains to be analyzed. The results show that the biological functions of RNAi systems and their relevance may differ markedly between different fungi, even among species of the same genus, and indicate that it is a very versatile tool that each fungus has uniquely adapted to its own needs.

## Figures and Tables

**Table 1:** Putative transposable elements detected in *F. fujikuroi* and *F. oxysporum* genomes.

Transposable Element	*F. fujikuroi*			*F. oxysporum*		
	Gene ID	Identity (%)	E-Value	Gene ID	Identity (%)	E-Value
HobS	*FFUJ_04535*	100	0	*FOXG_15494*	39.09	9 × 10^−142^
	*FFUJ_02576*	100	0	*FOXG_12541*	25.94	6 × 10^−65^
	*FFUJ_04521*	96.24	0	*FOXG_15105*	26.89	4 × 10^−57^
	*FFUJ_05678*	96.24	0	*FOXG_06872*	43.48	8 × 10^−49^
	*FFUJ_05881*	100	0	*FOXG_16138*	43.48	1 ×10^−48^
Impala	*FFUJ_07491*	87.35	0	chr14: 1236756-1237727 ^1^	89.56	0
	chrV: 2117840-2118331 ^1^	29.09	7 × 10^−13^	chr14: 1117562-1116699 ^1^	87.84	0
	-	-	-	chr14: 121747-121423 ^1^	87.80	1 × 10^−103^
	-	-	-	chr1: 6134797-6136077 ^1^	82.54	0.0
	-	-	-	chr15: 1546451-1545171 ^1^	82.54	0.0
Ty1/copia	*FFUJ_08062*	31.08	7 × 10^−66^	chr3: 342220-343926 ^1^	98.42	0.0
	*FFUJ_13355* ^2^	31.08	7 × 10^−66^	chr3: 415150-416856 ^1^	98.42	0.0
	*FFUJ_00408*	31.08	1 × 10^−65^	chr3: 982302-984008 ^1^	98.42	0.0
	*FFUJ_14407*	30.59	3 × 10^−65^	chr3: 3405691-3403985 ^1^	98.42	0.0
	-	-	-	*FOXG_14142*	43.42	6 × 10^−62^
Skippy	chrIV: 3140654-3142846 ^1^	53.08	0	chr5: 4489311-4493195 ^1,2^	97.99	0
	chrVI: 3662701-3660476 ^1^	52.96	0	DS231747: 67362-63478 ^1^	97.99	0
	chrIII: 4890426-4892675 ^1^	49.47	0	chr14: 1418859-1422395 ^1^	97.71	0
	chrX: 887601-889802 ^1^	52.04	0	*FOXG_17761*	36.73	2 × 10^−137^
	*FFUJ_00160*	25.77	6 × 10^−68^	*FOXG_17760*	40.37	9 × 10^−133^
MAGGY	chrX: 4392-6848	37.61	4 × 10^−134^	*FOXG_17760*	68.33	0.0
	*FFUJ_00160*	26.67	3 × 10^−78^	*FOXG_17761*	66.33	0.0

^1^ Unpredicted in the genome annotation. ^2^ Transposable elements shown in Figure 4.

**Table 2:** Features and genomic locations of precursor transcription sites for the de novo predicted milRNAs in the merged *F. fujikuroi* sRNA dataset.

ID	Score	Read Count	Mature milRNA 5′-3′ Sequence	Physical Location ^1^	Genetic Location	Gene Function
#1	1.3 × 10^1^	34	UGGGACGAGGACAAGGCUGAA	Chr VII 1735744	*FFUJ_08557* ORF Antisense orientation	Uncharacterized protein
#2	3.8	14	UCACCGUUAGACCAUUACAG	Chr VII 2455572	*FFUJ_08366* promoter 451 pb upstream SC ^2^ Sense orientation	Uncharacterized protein
#3	2.7	5	GUCCUGGAGGCACUUGA	Chr VII 1512406	*FFUJ_08631* ORF Sense orientation	Related to hydrolase
#4	2.3	2	GGCGCGAGAAGAGAUCGAGGAUC	Chr VII 868943	*FFUJ_02416* ORF Sense orientation	Related to nitrate reductase
#5	2.1	3	AGCCCAAUCCUUGUGCCACU	Chr II 1257164	*FFUJ_04898* ORF Antisense orientation	SRP40-Suppressor of mutant AC40 of RNA polymerase I and III
#6	1.9	3	AGAGGAAUCGACGAUGUGACU	Chr I 3119036	*FFUJ_01140* promoter 652 bp upstream from SC ^2^ Antisense orientation	Uncharacterized protein
#7	1.5	68	UGCAGAGCUUAUUCUAUCCC	Chr III 2168980	*FFUJ_02787* promoter 2117 bp upstream from SC ^2^ Sense orientation	Uncharacterized protein
#8	1.5	330	UUAGGGUUAGGGUUAGGGUUA	Chr X 2543622	Subtelomeric region	No gene
#9	1.4	2	UCCGAGCGCCAUGGUUGAUGAGA	Chr VII 2396236	*FFUJ_08382* ORF Antisense orientation	Uncharacterized protein
#10	0.6	25	UUCCACUACCUAUGGUCGUAU	Chr II 2372098	*EFFFUG00000000016* ORF Antisense orientation	
#11	0	5	UCGACAACCUCGUCUGCCUC	Chr IV 1640658	*FFUJ_13515* ORF Antisense orientation	Probable acetolactate synthase small subunit precursor

^1^ Chromosome and base position in the genomic sequence. ^2^ SC: start codon.

**Table 3:** Identity of candidate targets of some of the predicted milRNAs in *F. fujikuroi*.

ID	E-Value	Identity Sequence ^1^	Target Gene	Putative Function
#1	0.36	CGAGGACAAGGCTGA (AS)	*FFUJ_01736*	Related to plasma membrane phosphatase required for sodium stress response
#1	0.36	GAGGACAAGGCTGAA (S)	*FFUJ_09223*	Related to alpha-L-rhamnosidase A
#4	0.42	CGAGAAGAGATCGAG (S)	*FFUJ_02865*	Probable D-xylose reductase
#5	0.079	CCAATCCTTGTGCCAC (S)	*FFUJ_02776*	Related to cysteine-rich protein NFX-1
#7	0.079	TGCAGAGCTTAT[C]CTATCCC ^2^ (AS)	*FFUJ_13355* *FFUJ_14407* *FFUJ_00408*	Retrotransposon HobS hobase (*FFUJ_13355* and *FFUJ_14407*) TY2B-TY2B protein (*FFUJ_00408*)
#8	-	UUAGGGUUAGGGUUAGGGUUA	None	Telomeric DNA
#9	0.42	CGCCATGGTTGATGA (AS)	*FFUJ_01768*	Uncharacterized protein
#10	0.001	TTCCACTACCTATGGTCGT (AS)	rDNA *loci*	8 S ribosomal RNA (116 bases)
#11	0.31	AACCTCGTCTGCCTC (AS)	*FFUJ_13704*	Related to COG5-conserved oligomeric Golgi complex

^1^ In parentheses: sense (S) or antisense (AS) orientation of milRNA referred to the target. ^2^ In brackets: a non-pairing base.

## Data Availability

All sequencing files are available in the GEO repository under accession numbers GSE53159 (RNA-seq on the effect of dcl2 deletion) and GSE253160 (RNA-seq data for small RNAs).

## Supplementary Materials

The following supporting information can be downloaded at: https://www.mdpi.com/article/10.3390/ncrna10030031/s1, Figure S1: Expression at the genomic locations of the predicted milRNAs in the *F. fujikuroi* genome; Figure S2: Phenotypic characterization of Δ*dcl2* transformants; Figure S3: Dispersion and distribution graphs of RNA-seq samples from the wild-type strain and *dcl2* mutant; Figure S4: Effect of *dcl2* deletion on transcript levels (RPKM) in the dark for the nine *FFUJ_* genes in whose ORFs or putative promoter sequences the milRNAs shown in Table 2 are encoded; Figure S5: Effect of *dcl2* deletion on transcript levels (RPKM) in the dark of the putative milRNAs target *FFUJ_* genes described in Table 3 (A) and of the annotated *FFUJ_* genes for transposable elements described in Table 1 (B); Figure S6: Molecular analysis of the deletion of the *dcl2* gene in the *F. fujikuroi* wild-type strain; Table S1: Mapping results of sRNA sequencing; Table S2: Reads mapping to predicted ribosomal DNA; Table S3: Origin of sRNAs in *Fusarium*; Table S4: Formation of small RNAs within genomic features in sense orientation (dark vs. light); Table S5: De novo predicted miRNA-like RNAs in the merged sRNA dataset of *F. fujikuroi*; Table S6: Basic characteristics of the sequenced samples and yield of the readings; Table S7: Summary of the sequencing characteristics of each RNA-seq sample; Table S8: Primer sets used for PCR experiments.
