# Supplementary material for: The RNAi Machinery in the Fungus Fusarium fujikuroi Is Not Very Active in Synthetic Medium and Is Related to Transposable Elements
Source: Noncoding RNA. 2024 May 16;10(3):31. doi: 10.3390/ncrna10030031 (PMC11130915; doi:10.3390/ncrna10030031)
Supplement: Supplementary file 1 [file ncrna-10-00031-s001.zip › ncrna-2926465-supplementary.pdf]

Supplementary Material

The RNAi machinery in the fungus *Fusarium fujikuroi* is not very active in synthetic medium and is related to transposable elements

Javier Pardo-Medina, Tim A. Dahlmann, Minou Nowrousian,

M. Carmen Limón, and Javier Avalos

Supplementary Figures

Figure S1 a

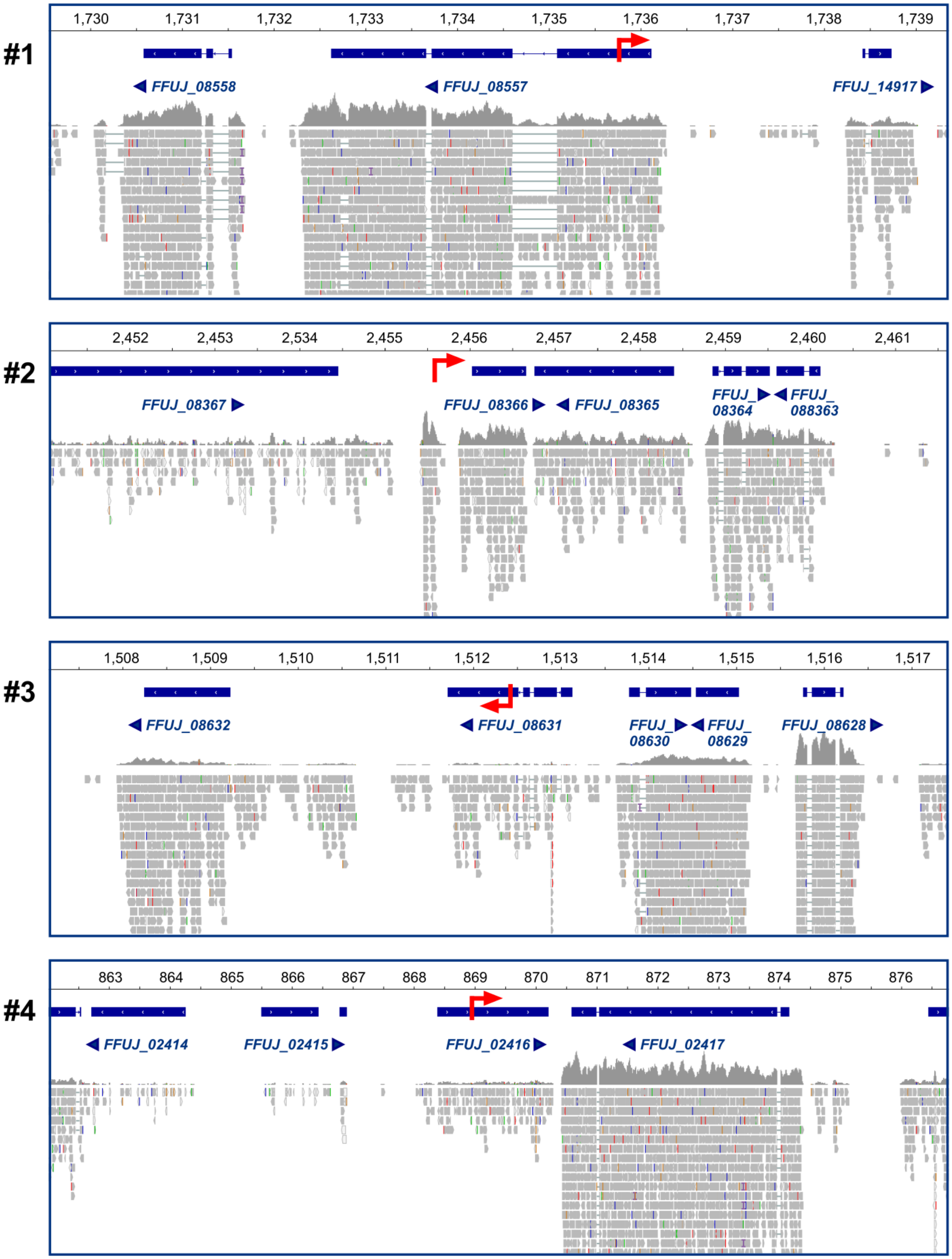

Figure S1 a (cont.)

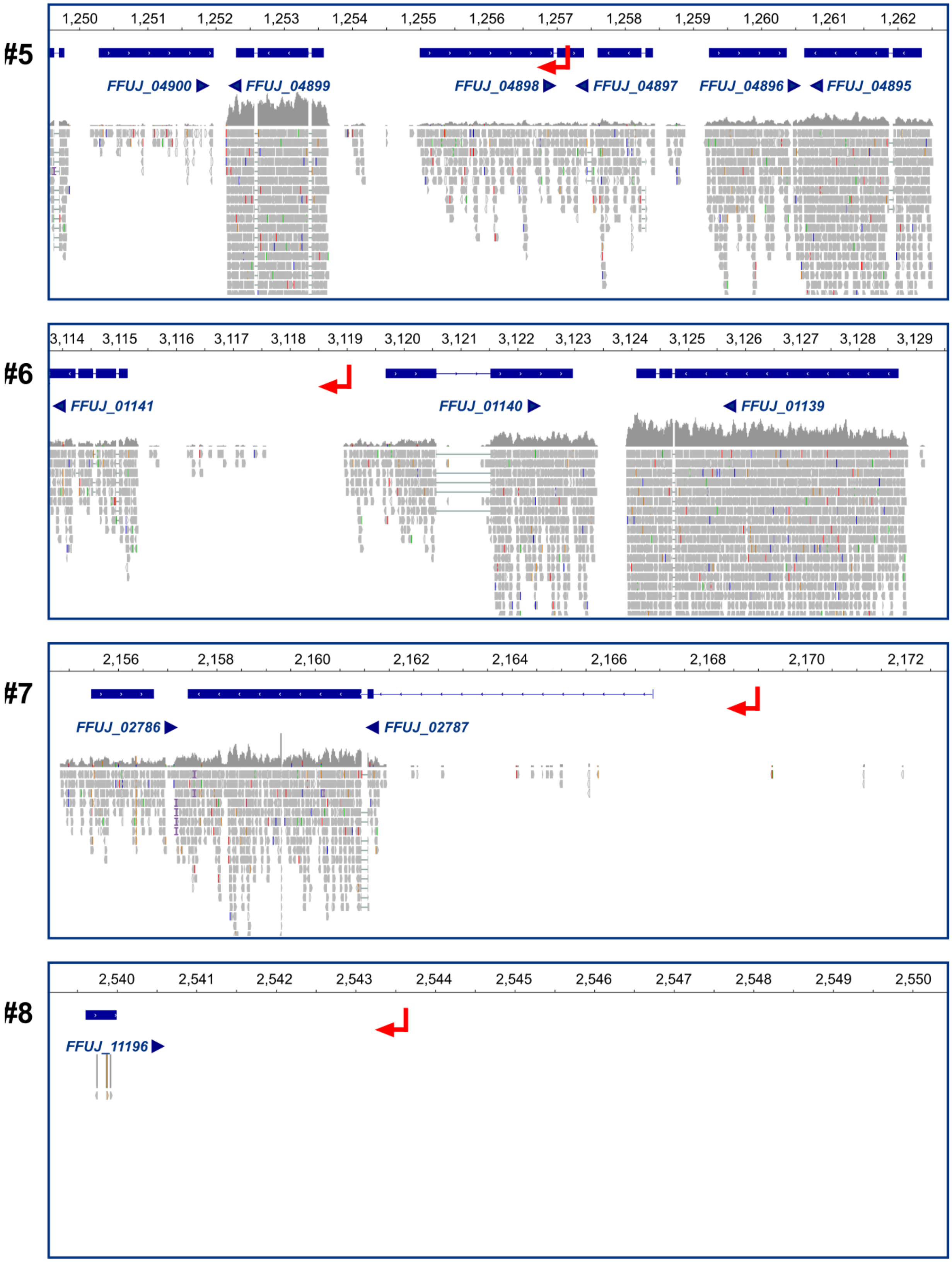

Figure S1 a (cont.)

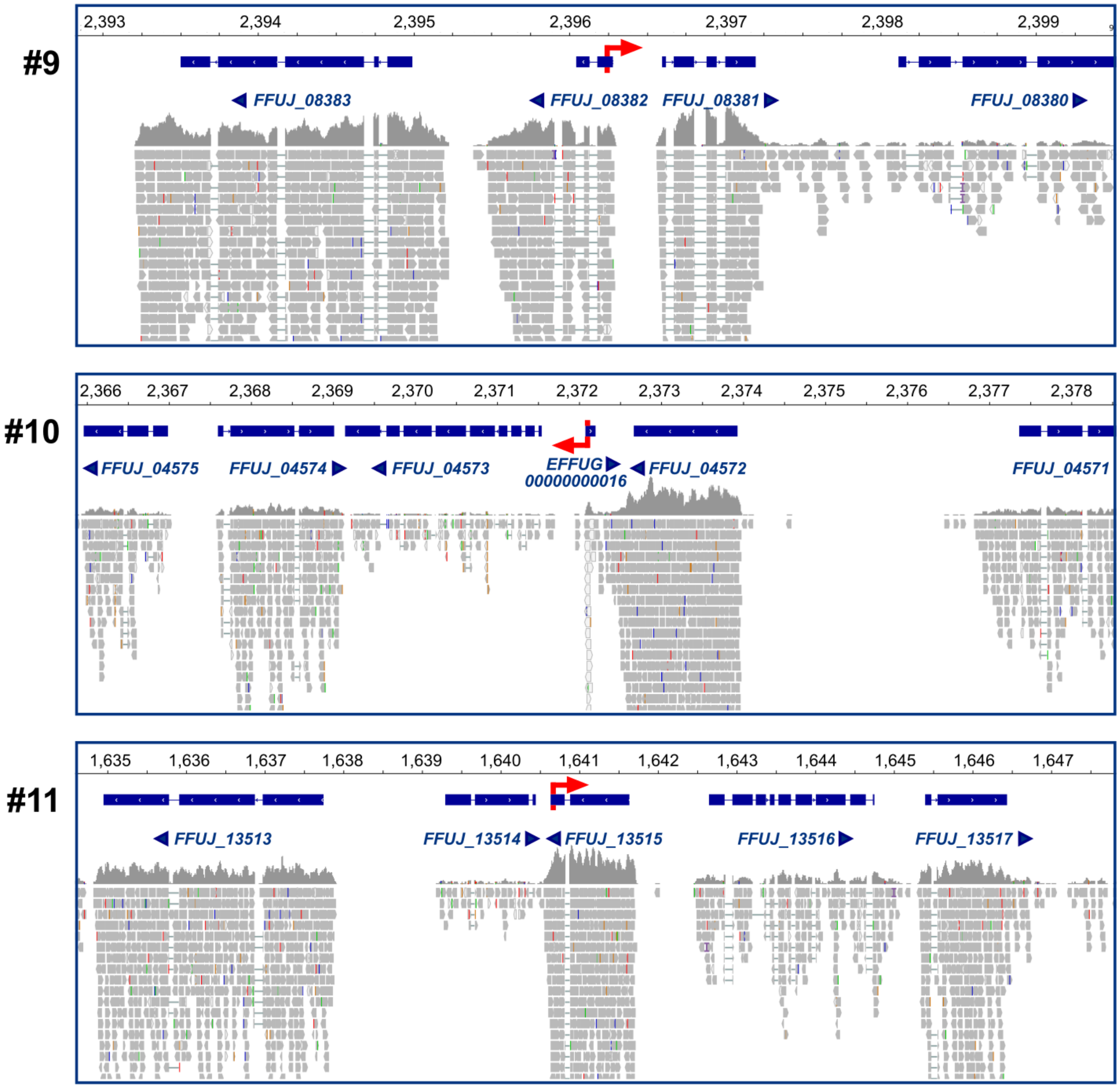

Figure S1 b

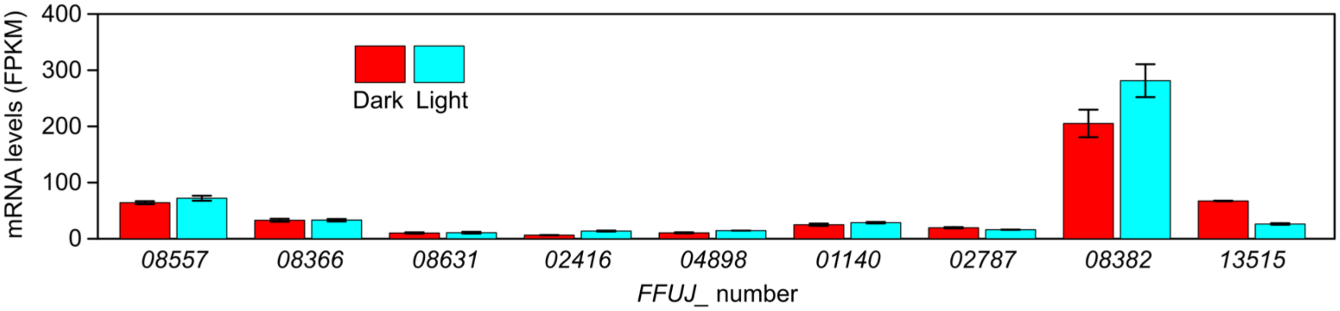

Figure S1. Expression at the genomic locations of the predicted milRNAs in the *F. fujikuroi* genome. (**a**) Transcript readings in the wild type in the genomic regions where the genes of the eleven predicted miRNA-like RNAs described in Table 2 are located. Gene names and orientation of the annotated genes are indicated in blue. Positions in kb for the chromosomes indicated in Table 3 are shown on top of each graph. The location and transcription sense of the predicted milRNAs are highlighted in red. Readings and genomic location viewed with the IGV program (Integrative Genomics Viewer, version 2.16). Readings correspond to one of the two samples from the wild type grown in the dark. For more information, see Figure 6. (**b**) Transcript levels (FPKM) in the wild type grown in the dark or after one hour illumination (light) of the nine *FFUJ_* genes in whose ORFs or putative promoter sequences are encoded the microRNAs shown in Table 2. RNA samples are the same from which the sRNAs were analyzed.

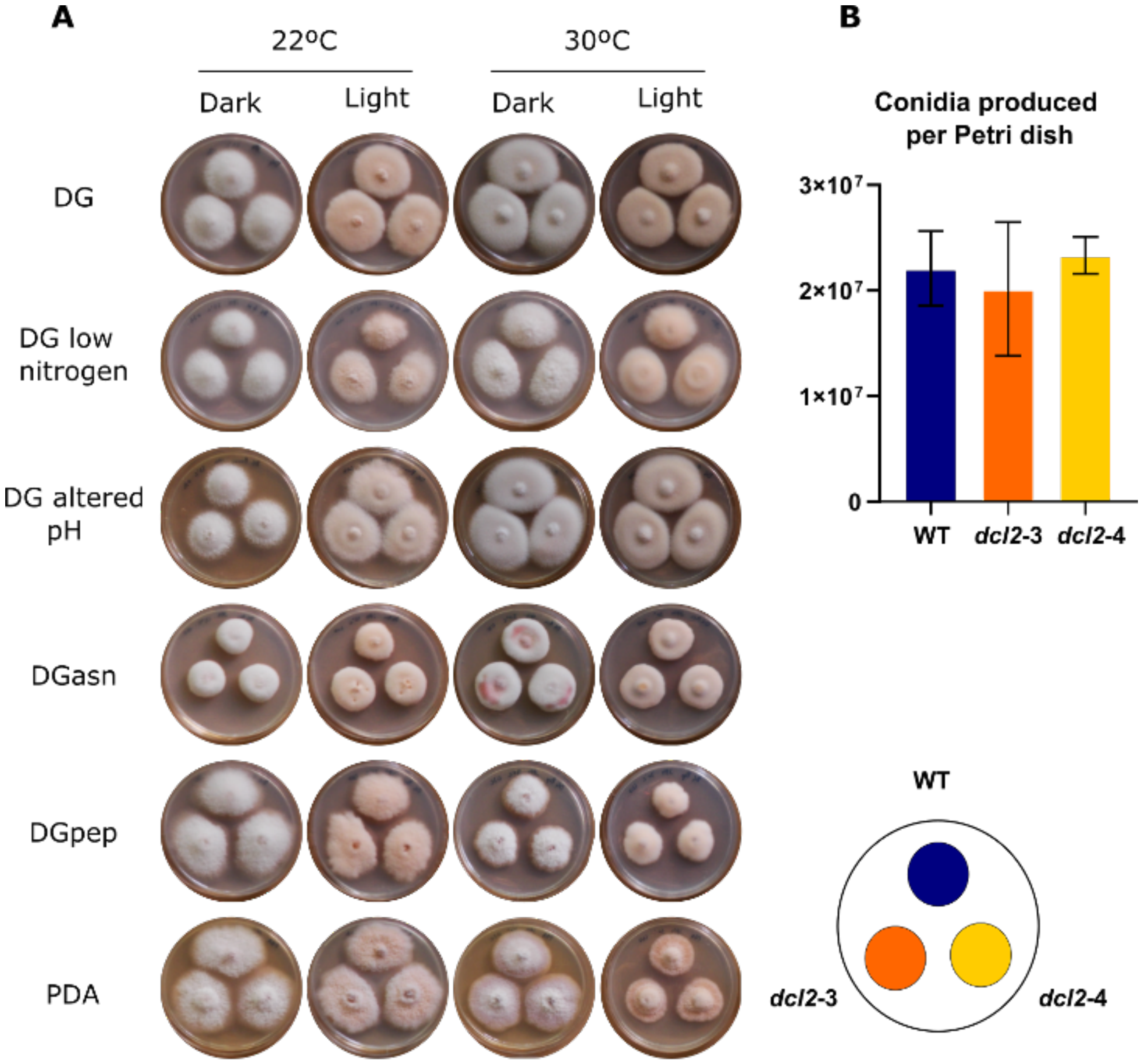

Figure S2. Phenotypic characterization of Δ*dcl2* transformants. (**a**) Growth and pigmentation of wild strain and Δ*dcl2* mutants *dcl2*-3 (SG293) and *dcl2*-4 (SG294) in the following media: DG: minimal medium, DG low nitrogen: DG with 0.3 g l^{-1} $NaNO_3$, DG with altered pH: DG with K_2HPO_4 (neutral pH) instead of H_2KPO_4 (acidic pH), DGasn: DG with asparagine instead of nitrate, DGpep: DG supplemented with 2 g l^{-1} peptone, PDA: potato dextrose agar. All strains were cultured per triplicate in each medium for one week at 22 °C or 30 °C, in the dark and under continuous illumination. Disposition of the three strains in the Petri dishes in the medium screening is shown on the right. (**b**) Conidia production of the wild strain and the two Δ*dcl2* mutants on EG medium. The values are the average of four independent determinations. The bar represents ± standard error.

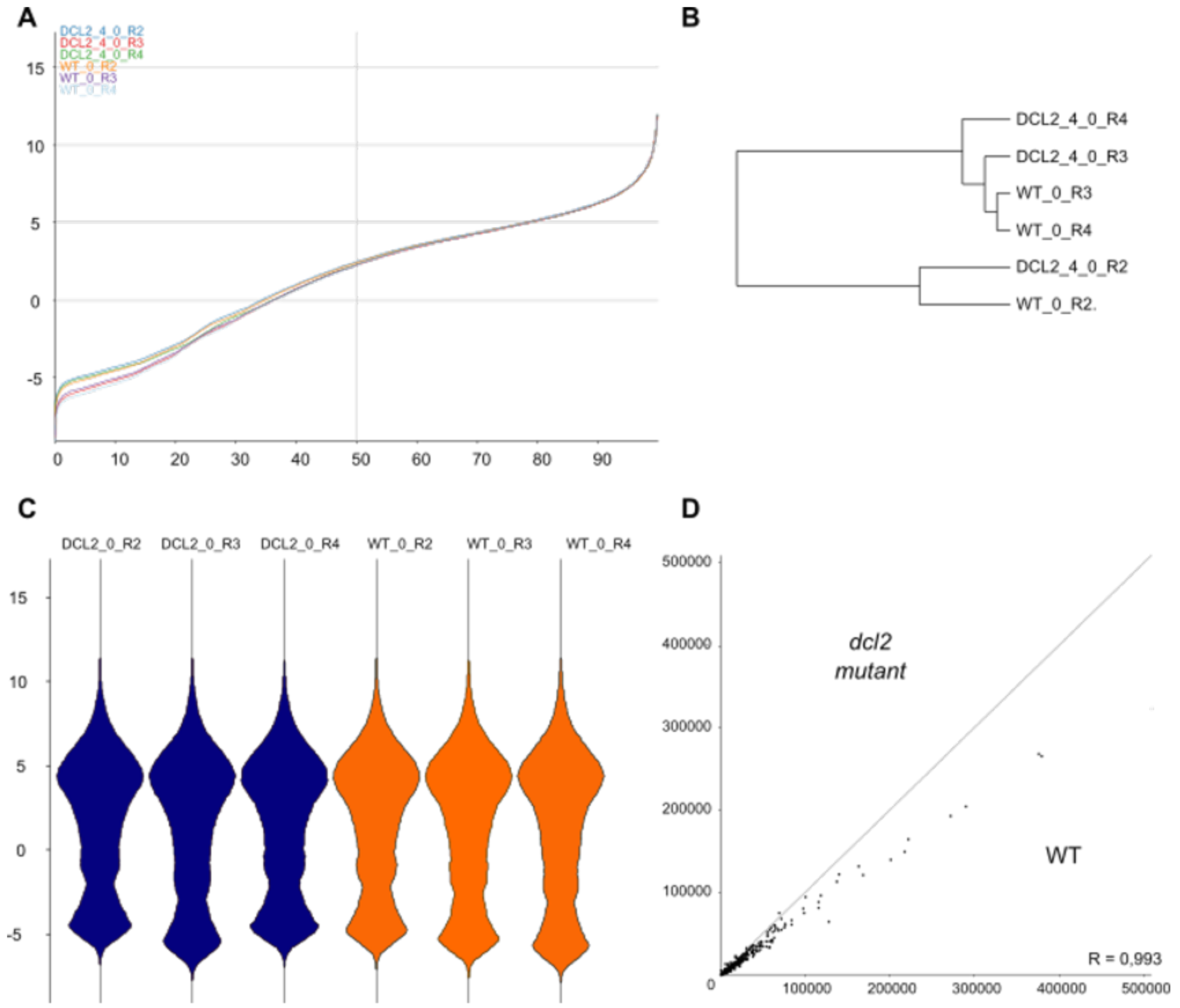

Figure S3. Dispersion and distribution graphs of RNA-seq samples from the wild-type strain and *dcl2* mutant. (**a**) Cumulative distribution plot after percentile normalization. The plot orders the probe values from lowest to highest, and then samples this set of values at common percentiles through the distribution. The x-axis therefore shows how far through the distribution we are looking, and the y-axis shows the value that the probe in that position has in that data store. (**b**) Neighbor joining tree calculated using a Pearson correlation to obtain a distance matrix between all the samples (rpm). (**c**) Bean distribution plots. (**d**) Scatter plot representation comparing the transcriptomes (log2 rpm values of each gene) of the wild-type strain (WT) and the Δ*dcl2* mutant.

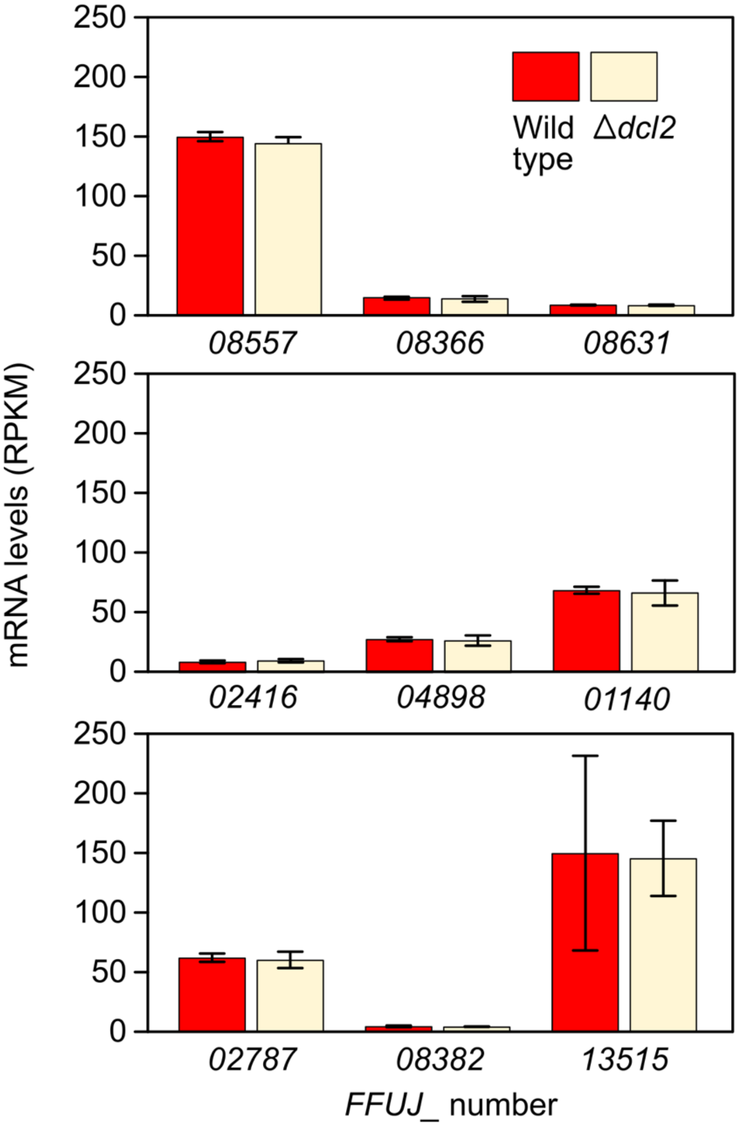

Figure S4. Effect of *dcl2* deletion on transcript levels (RPKM) in the dark for the nine *FFUJ_* genes in whose ORFs or putative promoter sequences are encoded the milRNAs shown in Table 2.

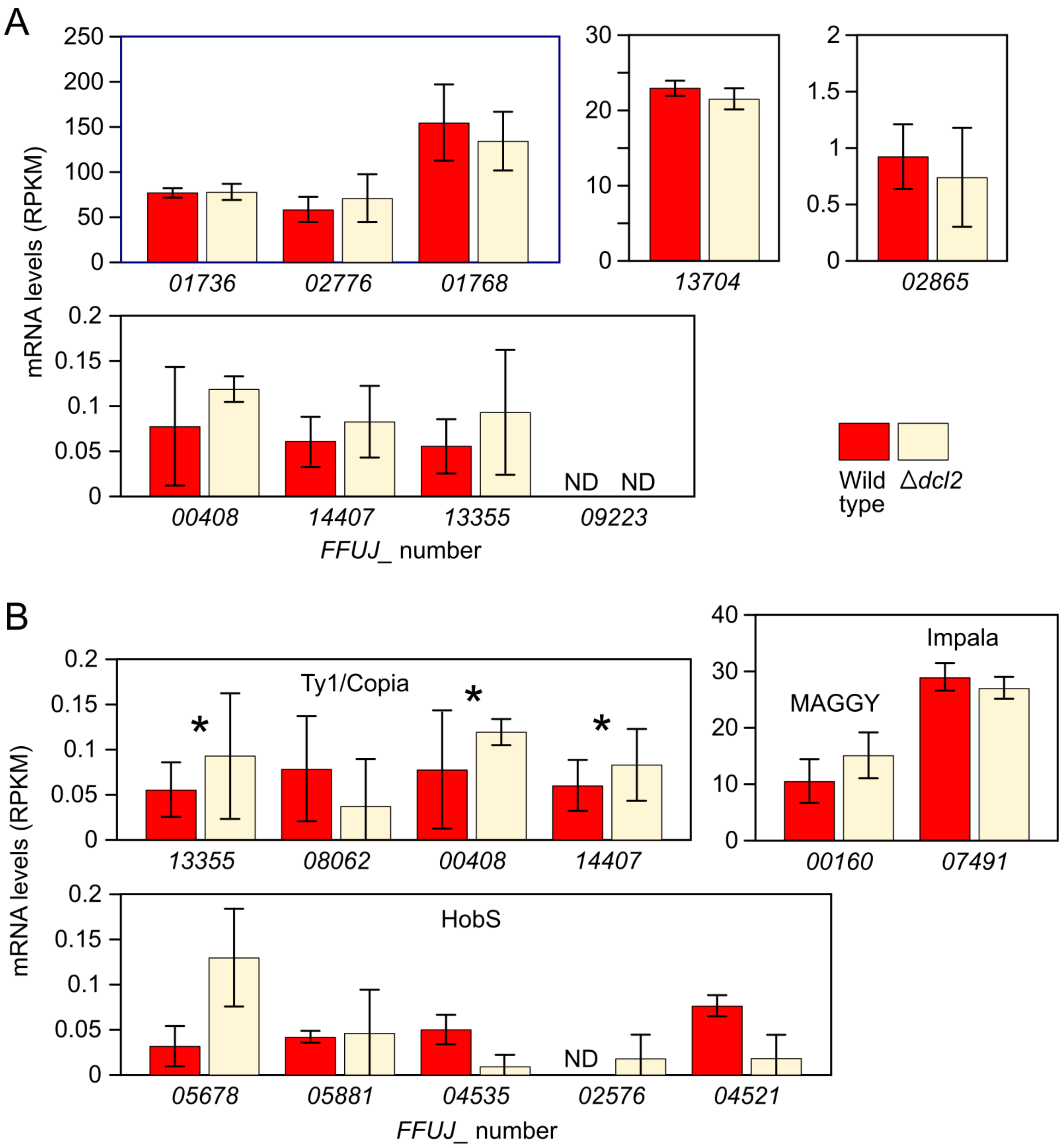

Figure S5. Effect of *dcl2* deletion on transcript levels (RPKM) in the dark of the putative milRNAs target *FFUJ_* genes described in Table 3 (**A**) and of the annotated *FFUJ_* genes for transposable elements described in Table 1 (**B**). The asterisk indicates three transposable elements found as possible targets of milRNAs in Table 3. ND: Non detected.

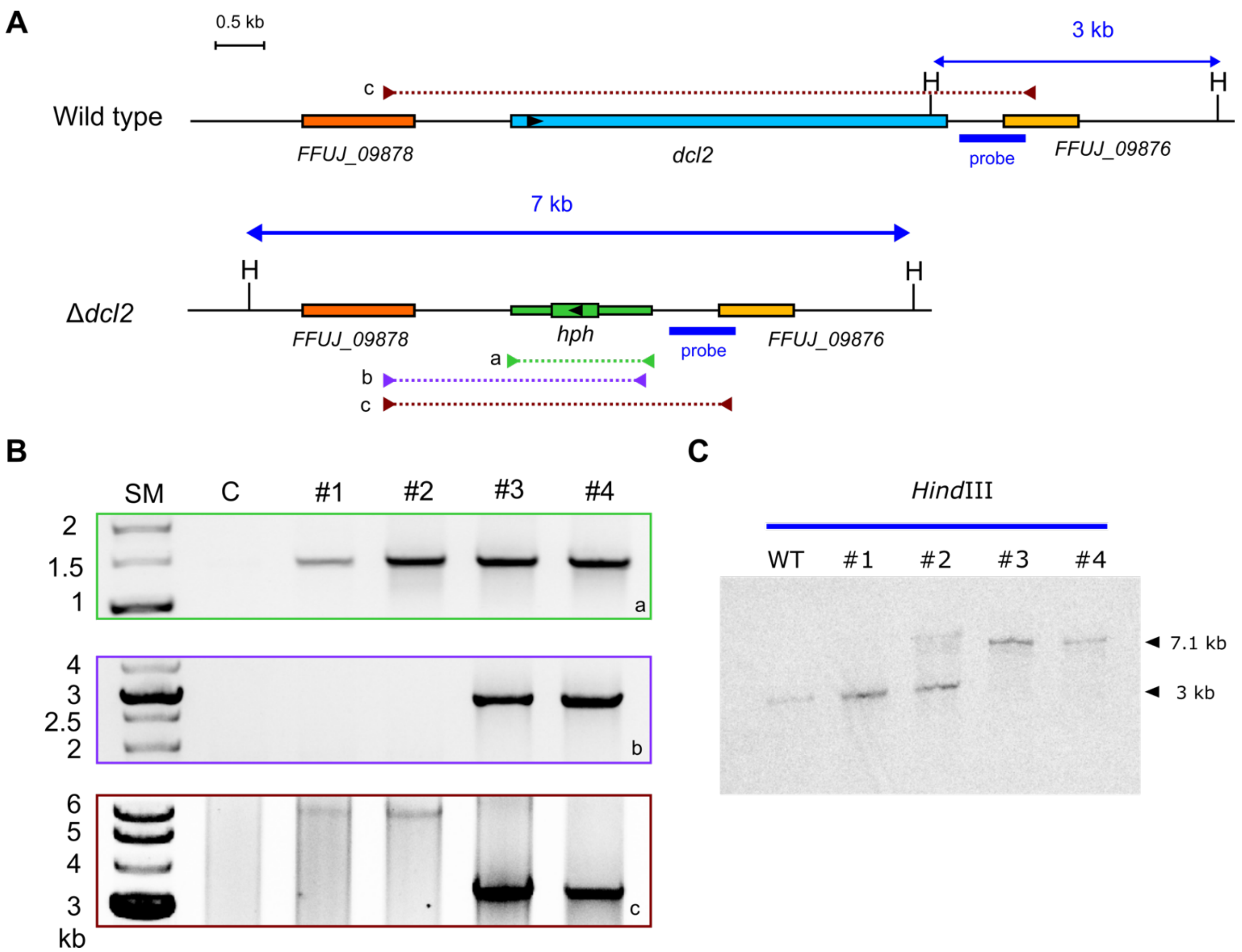

Figure S6. Molecular analysis of the deletion of the *dcl2* gene in the *F. fujikuroi* wild-type strain. (**a**) Genomic map covering the area for the wild strain (WT) and the Δ*dcl2* mutants showing the replacement with *hph* gene. (**b**) PCR amplifications of transformants #1, #2, #3, and #4 to select candidates with the correct replacement of *dcl2*. Primer sets (PS) used to amplify relevant regions are indicated on the map with colored arrowheads and their corresponding products are indicated with colored dotted lines. Expected band sizes in positive transformants for the different PCRs are 1,432 bp for amplicon a (in green) using PS3 primer set, 2,781 bp for amplicon b (purple) using PS4 primer set, and 3,660 bp for reaction c (red) using PS5 primer set. In the wild strain no amplification was expected for reactions a (green) and b (purple), while a band of 6,842 bp was expected for reaction c (red). (**c**) Southern blot of transformants #1, #2, #3, and #4 to test the correct integration. SM: Size markers. C: DNA-free control. *Hin*dIII restriction sites are indicated as H. Hybridization probe is indicated in the upper map as a blue bar and the expected hybridization products as blue lines, including the expected sizes of the bands in the Southern blot. The 769-pb probe was amplified with PS6 primer set.

Supplementary Tables

Table S1. Mapping results of sRNA sequencing.

Organism	Type of reads	Total reads	Mapped reads	Unmapped reads	Reads discarded by -m 5
F. fujikuroi	All reads	153,264,248	115,369,397 (75.3 %)	24,828,229 (16.2 %)	13,066,622 (8.5 %)
	Collapsed reads	6,612,908	4,447,147 (67.3 %)	2,105,318 (31.8 %)	60,443 (0.91 %)
	Collapsed reads (x≥10)	272,776	195,387 (71.6 %)	67,398 (24.7 %)	9,991 (3.7 %)
F. oxysporum	All reads	173,210,120	18,021,169 (10.4 %)	12,125,747 (7.0 %)	143,063,204 (82.6 %)
	Collapsed reads	7,034,397	3,920,202 (55.7 %)	2,096,047 (29.8 %)	1,018,148 (14.5 %)
	Collapsed reads (x≥10)	305,475	76,281 (25.0 %)	51,981 (17.0 %)	177,213 (58.0 %)

Table S2. Reads mapping to predicted ribosomal DNA.

Organism	Type of reads	Total reads	Reads mapping to rDNA	Unmapped reads
F. fujikuroi	Total reads	153,264,248	25,029,046 (16.3 %)	128,235,202 (83.7 %)
	Collapsed reads	6,612,908	251,425 (3.8 %)	6,361,483 (96.2 %)
F. oxysporum	Total reads	173,210,120	127,479,642 (73.6 %)	45,730,478 (26.4 %)
	Collapsed reads	7,034,397	866,926 (12.3 %)	6,167,471 (87.7 %)
F. fujikuroi (incl. rDNA from *F. oxysporum*)	Total reads	153,264,248	107,751,205 (70.3 %)	45,513,043 (29.7 %)
	Collapsed reads	6,612,908	740,534 (11.2 %)	5,872,374 (88.8 %)

Table S3. Origin of sRNAs in *Fusarium*. Relevant data are marked in red. Predicted rDNAs of both *Fusarium* species were used for calculation of rRNA-mapping reads in *F. fujikuroi* and *F. oxysporum*.

		F. fujikuroi			***F. oxysporum***		
Feature	Strand	Total reads	Collapsed reads	Collapsed reads (x≥10)	Total reads	Collapsed reads	Collapsed reads (x≥10)
rRNA	Sense	107,441,729	734,758	137,200	132,061,732	894,041	162,931
	Antisense	1,438,898	53,471	7,023	2,495,057	25,796	3,250
tRNA	Sense	10,061,987	42,861	6,378	8,668,518	49,186	7,132
	Antisense	70,482	3,372	34	100,435	3,192	17
CDS	Sense	5,911,950	2,759,531	251,402	5,763,287	2,504,352	11,297
	Antisense	7,978,889	243,429	14,888	2,563,605	182,700	1,322
Intron	Sense	2,719,506	104,448	6,982	2,555,759	122,882	7,165
	Antisense	7,712,652	39,037	2,584	4,649,363	94,585	9,982
Intergenic	Both	111,807,186	1,633,420	155,047	153,725,629	2,493,370	234,515

Table S4. Formation of small RNAs within genomic features in sense orientation (dark *vs*. light). The listed genes show a downregulation of log2 < -1 and an adjusted *p*-value < 0.1.

Gene ID	Functional annotation	Base Mean	log2FC	lfcSE	stat	P-value	padj
FFUJ_08272	uncharacterized protein	349.39	-4.14	0.37	8.39	4.94E-17	1.76E-13
FFUJ_11803	*carB*	626.88	-4.01	0.37	8.19	2.58E-16	4.60E-13
FFUJ_11804	*carO*	318.30	-3.95	0.38	7.75	9.21E-15	1.09E-11
FFUJ_01292	uncharacterized protein	329.32	-3.84	0.37	7.57	3.87E-14	3.44E-11
FFUJ_09320	related to Rds1 protein	230.73	-3.68	0.38	7.02	2.22E-12	1.58E-09
FFUJ_06055	*vvdA*	267.11	-3.57	0.38	6.79	1.11E-11	6.57E-09
FFUJ_13896	related to TGF beta induced protein ig-h3 precursor	461.74	-3.45	0.37	6.63	3.32E-11	1.69E-08
FFUJ_01088	related to short-chain alcohol dehydrogenase	913.82	-3.21	0.38	5.85	4.99E-09	2.22E-06
FFUJ_11802	*carRA*	245.30	-3.12	0.37	5.67	1.39E-08	4.97E-06
FFUJ_12435	uncharacterized protein	87.71	-3.12	0.40	5.35	9.01E-08	2.92E-05
FFUJ_04335	uncharacterized protein	1042.24	-3.05	0.36	5.68	1.38E-08	4.97E-06
FFUJ_09119	related to flavin-containing amine oxidasedehydrogenase	114.61	-2.80	0.39	4.58	4.61E-06	1.37E-03
FFUJ_11801	*carX*	76.47	-2.66	0.40	4.18	2.94E-05	8.04E-03
FFUJ_05732	*cryD*	169.08	-2.52	0.39	3.92	9.03E-05	2.14E-02
FFUJ_08014	related to formaldehyde dehydrogenase	97.23	-2.50	0.39	3.83	1.27E-04	2.83E-02
FFUJ_05515	probable ATP-binding multidrug cassette transport protein	1024.56	-2.46	0.36	4.09	4.28E-05	1.09E-02
FFUJ_00295	*con10*	110.77	-2.37	0.39	3.51	4.53E-04	9.39E-02
FFUJ_07515	related to arabinose 5-phosphate isomerase	145.09	-2.34	0.38	3.49	4.74E-04	9.39E-02

Table S5. De novo predicted miRNA-like RNAs in the merged sRNA dataset of *F. fujikuroi*.

ID [1]	Score	Read count: Total	Mature	Loop	Star	p-value	Mature sequence	Precursor sequence (mature sequence underlined)
VII_86570 #1	1.3e+1	34	33	0	1	no	UGGGACGAGGACAAGGCUGAA	UGGGACGAGGACAAGGCUGAAUGGGGGUUUAUGGUGGAAGGAUUGUUGGCGCUCGCAU
VII_90055 #2	3.8	14	12	0	2	no	UCACCGUUAGACCAUUACAG	UAUUGGGAUGGGCGGUUGAGCGGGUUUGAACGCCUUCACCGUUAGACCAUUACAG
VII_98350 #3	2.7	5	4	0	1	yes	GUCCUGGAGGCACUUGA	CGAGUAUACUUUGGUGCCUGAUCAAGUUUACCCAAGGCAGGUCCUGGAGGCACUUGA
III_209346 #4	2.3	2	1	0	1	yes	GGCGCGAGAAGAGAUCGAGGAUC	CCGGCAGAUCUCGUCGACGGGCGACCGGCGCGAGAAGAGAUCGAGGAUC
II_193099 #5	2.1	3	2	0	1	yes	AGCCCAAUCCUUGUGCCACU	AGCCCAAUCCUUGUGCCACUCACUAUGACACUGGUGGCAUCCUCUCCCCGGGUUGUGAGGACAGGGAUGAACCU
I_289987 #6	1.9	3	2	0	1	yes	AGAGGAAUCGACGAUGUGACU	AGAGGAAUCGACGAUGUGACUUUGGCGUCAAAGGUUGGUAGGUUGGCGUCAAAAUGCGUUGUCGUACCUGAGGA
III_235482 #7	1.5	68	42	26	0	yes	UGCAGAGCUUAUUCUAUCCC	UGCAGAGCUUAUUCUAUCCCUUUAGGCCUCCCGCUUUCCUGCACUGGAUUGGUUUAGAGGCUAAGGUAAGCUCCCUCUU
X_17800 #8	1.5	330	330	0	0	yes	UUAGGGUUAGGGUUAGGGUUA	GCCUCUUACCUUCCCCGAUUAAACGAAAAUCUUGCGUUUUGCCUUAGGGUUAGGGUUAGGGUUA
VII_89770 #9	1.4	2	1	0	1	yes	UCCGAGCGCCAUGGUUGAUGAGA	UCUUGACCGUGGCUUUGGGGUAUGGUUUCUUUCCGAGCGCCAUGGUUGAUGAGA
II_198003 #10	0.6	25	25	0	0	yes	UUCCACUACCUAUGGUCGUAU	UUCCACUACCUAUGGUCGUAUGUACCUAUUGACUAUGGGAAGAGGAUAA
IV_60042 #11	0	5	4	0	1	no	UCGACAACCUCGUCUGCCUC	UCGACAACCUCGUCUGCCUCCAUGACAAGGGACUCCUGGUUACCUUCAGACAGAGGAGAUCGGGGUAGAGCC

[1] Chromosome and reference number.
Below, identification number used in the main text.

Table S6. Basic characteristics of the sequenced samples and yield of the readings.

Sample	Number of sequences	Average length	Average quality	G+C (%)	Mapping rate (%)
WT.0.R2	24235008	75.35	36.28	52	98.48
WT.0.R3	34924904	75.37	36.31	52	98.68
WT.0.R4	47559597	75.35	36.29	52	98.70
dcl2.4.0.R2	20277079	75.29	36.28	52	98.61
dcl2.4.0.R3	39929806	75.29	36.26	52	98.51
dcl2.4.0.R4	21674473	75.40	36.32	52	98.68

Table S7. Summary of the sequencing characteristics of each RNA-seq sample.

	Sample name	Number of trimmed sequences	Average length	Average quality	Sequences 18-25 nt (%)
F. fujikuroi	Ffuj_dark_R1	47609550	38.84	39.41	11.97
	Ffuj_dark_R2	26679687	35.57	39.33	19.09
	Ffuj_light_R1	52755629	35.6	39.31	18.96
	Ffuj_light_R2	26219382	39.8	39.4	7.68
F. oxysporum	Foxy_dark_R1	32247420	35.17	39.32	19.07
	Foxy_dark_R2	46332088	33.8	39.33	21.10
	Foxy_light_R1	47081719	37.98	39.39	10.33
	Foxy_light_R2	47548893	39.45	39.3	7.30

Table S8. Primer sets used for PCR experiments.

Primer set	Primer names	5′-3′ Sequence	Experimental use
PS1	Ff-dcl2-pRS246-F	GTAACGCCAGGGTTTTCCCAGTCACGACGTGGC TATCTGTGATTTTAGTGTAC	PCR 5′ *dcl2* segment of pDcl2hyg
	Ff-dcl2-hph-R	ATCCACTTAACGTTACTGAAATCTCCAACCATT TTCCTATCATGGGGGAG	
PS2	Ff-dcl2- pRS246-R	GCGGATAACAATTTCACACAGGAAACAGCCCA AAGTCGATGCCGCTCT	PCR 3′ *dcl2* segment pDcl2hyg
	Ff-dcl2-hph-F	CTCCTTCAATATCATCTTCTGTCTCCGACGAAG GGGATCATGTACACGC	
PS3	HPH-6F	GTCGGAGACAGAAGATGATATTGAAGGAGC	PCR Hyg^R cassette
	HPH-6R	GTTGGAGATTTCAGTAACG TTAAGTGGAT	
PS4	Ff-predcl2-1F	CTCTTGTTGGCTTTCCATGCCG	PCR test 5′*dcl2* - HygR
	HPH-6F	GTCGGAGACAGAAGATGATATTGAAGGAGC	
PS5	Ff-predcl2-1F	CTCTTGTTGGCTTTCCATGCCG	PCR test *dcl2* - HygR
	Ff-postdcl2-1R	CTTCTTGCGCACGACATACGAG	
PS6	Ff-postdcl2-1F	GGAAGGCACCCTAACTGAGAACTC	Southern probe of *dcl2*
	Ff-postdcl2-1R	CTTCTTGCGCACGACATACGAG	
